# Transcriptomic Analysis Insights into Salt Adaptation of Pickle-Derived *Aspergillus westerdijkiae*

**DOI:** 10.3390/microorganisms14081778

**Published:** 2026-08-12

**Authors:** Xuelan Liao, Bo Song, Zhen He, Tingfu Zhang, Guoqin Wen

**Affiliations:** 1 College of Life Sciences, China West Normal University, Nanchong 637009, China; xinyunzj@126.com (X.L.); songbo1@126.com (B.S.); 2 Nanchong Fruit Tree Guidance Station, Nanchong 637000, China; nchezhen@hotmail.com

**Keywords:** *Aspergillus westerdijkiae*, salt stress, HOG-MAPK pathway, glycerol metabolism, ion homeostasis, transcriptome, lipid recycling

## Abstract

*Aspergillus westerdijkiae*, a filamentous fungus commonly isolated from pickled vegetables and high-salt condiments, can cause spoilage and produce nephrotoxic ochratoxin A (OTA) under saline conditions. However, its adaptive mechanisms to salt stress remain unclear. To address this, the pickle-derived strain NDX1 was subjected to 0, 1.0, 1.5, and 2.0 mol/L NaCl treatments. Colony growth was assessed after 7 days of incubation on PDA plates supplemented with the respective NaCl concentrations. For physiological indices, mycelia were pre-cultured in salt-free PDB for 5 days, followed by the addition of NaCl to final concentrations (0, 1.0, 1.5, and 2.0 mol/L) and further incubation for 2 days, after which relative electrical conductivity (REC) and malondialdehyde (MDA) content were measured. Colony diameters were recorded to evaluate vegetative growth; REC was determined by conductometry to assess cell membrane permeability; and MDA content was measured via the thiobarbituric acid (TBA) colorimetric method to indicate lipid peroxidation levels. Transcriptome sequencing combined with qRT-PCR validation was employed to identify differentially expressed genes (DEGs) involved in osmotic adaptation. Results showed that low salinity (1.0 mol/L NaCl) promoted growth, while higher concentrations (≥1.5 mol/L NaCl) inhibited it, accompanied by increased REC and decreased MDA, forming a distinctive high-permeability, low-lipid-peroxidation phenotype. A total of 3155 DEGs were detected, mainly associated with the HOG-MAPK cascade, glycerol biosynthesis, and ion transport pathways. Eight key HOG-MAPK genes and 21 glycerol metabolic genes were upregulated in a concentration-dependent manner, with the terminal kinase Hog1 coordinating transcription of downstream effectors governing glycerol synthesis and ion homeostasis. These findings demonstrate that *A. westerdijkiae* integrates *de novo* glycerol production and intracellular lipid remodeling via the HOG-MAPK pathway to achieve osmotic adaptation under hypersaline stress. This work identifies potential molecular targets for controlling toxigenic spoilage caused by this species in high-salt fermented foods.

## 1. Introduction

*Aspergillus westerdijkiae* is a dominant filamentous fungal contaminant widely isolated from fermented pickles, cured meat and high-salt condiments [1]. This species exhibits extraordinary halotolerance and can maintain vigorous vegetative growth under high NaCl environments that strongly inhibit most microbial competitors, making it a persistent spoilage agent in salt-rich fermented food systems [2]. Traditional high-salt preservation strategies rely on elevated sodium concentrations to suppress microbial proliferation; however, halotolerant filamentous fungi, including *A. westerdijkiae*, have evolved sophisticated adaptive regulatory networks to counteract hyperosmotic damage, which severely weakens the bacteriostatic and fungistatic effects of brine treatment [3]. Divergent plasma membrane remodeling strategies have been identified across extremophilic fungi, laying a critical physiological foundation for understanding salt stress adaptation at the cellular level [4].

This environmental selection is particularly relevant to pickled mustard production, where raw materials are treated with high concentrations of sodium chloride (typically 7–10%, *w*/*v*) during natural fermentation [1]. While high-salt treatment efficiently inhibits the proliferation of most bacterial contaminants, it simultaneously forms a unique selective microhabitat suitable for the survival and reproduction of halotolerant spoilage fungi such as *A. westerdijkiae* [5]. This fungus may contaminate products at multiple stages of the production chain, including raw material cleaning, manual processing and post-fermentation packaging. Its strong adaptability to hypersaline environments enables massive mycelial expansion on pickled products, resulting in visible fungal plaques, surface discoloration and soft texture deterioration of finished pickles. More importantly, *A. westerdijkiae* acts as a potent ochratoxin A (OTA) producer [1,5]. OTA exhibits prominent nephrotoxicity, immunotoxicity and carcinogenicity to humans and animals [6]. Due to its high thermal stability, OTA cannot be eliminated by routine post-processing procedures, and thus continuously threatens the safety of high-salt fermented vegetable products [7]. Despite the widespread contamination risk and toxic hazards caused by this strain, the molecular regulatory network supporting its robust salt tolerance has not been systematically clarified, restricting the establishment of targeted inhibition and control technologies for pickle spoilage fungi.

Salt tolerance is a core ecological trait that governs the colonization, expansion, and long-term survival of food-borne *Aspergillus* strains under hypersaline microhabitats [5,8]. The conserved high-osmolarity glycerol (HOG) MAPK signaling cascade acts as the master regulatory hub for fungal osmotic perception and downstream adaptive reprogramming across salt-adapted eukaryotes [9]. In the model filamentous fungus *A. nidulans*, upstream osmotic signals are exclusively transmitted via two-component histidine kinase modules to activate the HOG kinase cascade [10]. Distinct osmotic responses are triggered by different osmotic solutes, such as NaCl and mannitol, indicating that fungi deploy tailored metabolic strategies to cope with diverse hypertonic environments [11]. Glycerol serves as the primary compatible solute mitigating intracellular dehydration under salt exposure; the synthetic, catabolic, and transmembrane transport pathways of glycerol have been systematically characterized in yeast models [12,13]. Salt stress inevitably impairs plasma membrane integrity, and relative electrical conductivity (REC) has become a standard physiological biomarker to quantify the severity of membrane leakage induced by hyperosmotic injury [14]. Comprehensive reviews have summarized the conserved and divergent functions of the HOG-MAPK pathways across pathogenic *Aspergillus* species, providing a universal research framework for dissecting osmotic signaling in food-derived strains [15].

Recent transcriptomic investigations on the halotolerant model fungus *A. sydowii* revealed that the sugar transporter AsSTL is transcriptionally controlled by Hog1 to coordinate intracellular osmotic equilibrium [16]. Cross-talk between nutritional signal sensing and osmotic stress cascades extensively reshapes global gene transcription in filamentous fungi under abiotic pressure [17]. RNA-seq profiling of salt-challenged *A.* nidulans uncovered calcineurin-independent salt-resistance transcriptional branches, while deep transcriptome sequencing of salinity-treated *A. oryzae* further uncovered large sets of differentially expressed genes involved in cation trafficking and carbon metabolism [18,19]. Lipid metabolism reprogramming constitutes an indispensable adaptive strategy for fungi confronting high-salt stress: storage lipid breakdown and lipase-mediated lipid recycling supply supplementary carbon skeletons for osmoprotectant synthesis, while simultaneously alleviating salt-triggered lipid peroxidation [20]. Transcriptomic profiling of salt-adapted fermentative yeast and edible macrofungi further verified that salinity induces extensive remodeling of carbohydrate and lipid metabolic pathways [21,22]. Lactic acid bacterial metabolites interfere with fungal fatty acid and purine metabolism to inhibit the proliferation of spoilage fungi, indirectly reflecting the central role of lipid homeostasis in fungal salt adaptation [23].

Multi-omics profiling of *A.* sydowii cultivated under saturated NaCl revealed integrated adaptive mechanisms covering mycelial morphology, compatible solute accumulation, and oxidative stress mitigation [6]. Species-specific osmolyte signatures are formed in halophilic aspergilli to offset osmotic pressure and reduce cellular damage under hypersaline culture [24]. The biosynthesis-related gene cluster of secondary metabolites has been clarified in closely related food-borne *Aspergillus* species, which provides a reference for fungal stress-associated metabolic research [7]. Yeast relies on precise cation transport systems to maintain stable intracellular K^+^/Na^+^ ratios and relieve ionic toxicity under high-salt conditions, which provides a vital reference for studying ion homeostasis in filamentous fungi [25].

Although previous physiological and multi-omics studies have advanced our understanding of halotolerant aspergilli, three critical knowledge gaps remain unaddressed for pickle-isolated *A. westerdijkiae* NDX1: (1) the concentration-dependent activation mode of the complete HOG-MAPK cascade has not been systematically characterized; (2) the transcriptional coordination between Hog1, glycerol anabolism, and transmembrane ion transport remains unclear; (3) the synergistic regulatory network linking plasma membrane integrity, lipid recycling, and osmoprotectant production lacks integrated physiological and omics validation.

In the present work, the pickle-derived halotolerant isolate *A. westerdijkiae* NDX1 was cultivated under four gradient NaCl concentrations: 0, 1.0, 1.5, and 2.0 mol/L. Supplementary phenotypic observation and physiological index detection were carried out as auxiliary characterization, while whole-transcriptome sequencing served as the core approach to identify salt-responsive differentially expressed genes (DEGs). We mainly focused on dissecting transcriptional profiles of the HOG-MAPK cascade, glycerol biosynthesis, intracellular lipid recycling, and cation transport. Based on transcriptomic evidence combined with supporting physiological data, this work constructs a multi-layered osmotic regulatory network for pickle-isolated *A. westerdijkiae*, clarifies the core molecular mechanisms underlying its robust salt tolerance, and delivers theoretical foundations for developing targeted prevention strategies against toxigenic halotolerant fungi in high-salt fermented vegetable products.

## 2. Materials and Methods

### 2.1. Strain

*A*. *westerdijkiae* NDX1 was isolated from naturally fermented pickled mustard and taxonomically identified via morphological observation and molecular sequencing.

Routine strain activation was performed on potato dextrose agar (PDA) poured into standard plastic Petri dishes and incubated at 28 ± 1 °C. Liquid cultures for physiological tests and transcriptome sampling were cultivated in triangular flasks using potato dextrose broth (PDB). The formulation of 1 L PDA medium consists of 200 g potato infusion, 20 g D-glucose and 18 g agar. PDB adopts the same basal nutrient composition as PDA, with agar omitted.

For long-term cryopreservation, 15–20 loops of conidia were scraped from activated PDA plates and eluted into 700 μL sterile water and fully mixed. Afterwards, 300 μL analytically pure glycerol was added and homogenized thoroughly to reach a final glycerol concentration of 30%. The prepared spore-glycerol suspension was stored at −80 °C for strain backup.

### 2.2. Vegetative Growth Under Salt Stress

Spores from activated cultures were eluted with sterile distilled water to prepare a spore suspension at a final concentration of 1 × 10^6^ CFU/mL. A total of 150 μL of spore suspension was evenly spread onto PDA plates for sporulation. Mycelial plugs (7 mm in diameter) were punched with a sterile cork borer and placed mycelium-side downward onto a PDA medium supplemented with 0, 1.0, 1.5, or 2.0 mol/L NaCl, respectively. All plates were incubated at 28 ± 1 °C for 7 d, and colony diameters were measured using the perpendicular crossing method.

### 2.3. Determination of Mycelial Physiological Indices Under Salt Stress

#### 2.3.1. Salt Stress Treatment

A total of 200 μL of spore suspension (1 × 10^6^ CFU/mL) was inoculated into 100 mL potato dextrose broth (PDB). The culture was incubated at 28 °C with shaking at 150 rpm for 5 d. Then, solid NaCl was supplemented to the broth to achieve final concentrations of 0, 1.0, 1.5, and 2.0 mol/L, respectively. After another 2 days of incubation, mycelia were collected for the quantification of relative electrical conductivity (REC) and malondialdehyde (MDA) content.

#### 2.3.2. Relative Electrical Conductivity (REC) Measurement

REC was measured with slight modifications to the method described by Liu et al. [26]. Briefly, 0.5 g fresh mycelia pellets were rinsed twice with sterile water and surface-dried with filter paper. Samples were fully ground in liquid nitrogen and transferred into 15 mL of deionized water, followed by a 30 min water bath at 25 °C. The initial conductivity was recorded as R_1_. Subsequently, the mixture was heated in a boiling water bath for 30 min to completely rupture cells. After cooling to ambient temperature, the final conductivity was measured and defined as R_2_.

REC (%) = (R_1_/R_2_) × 100% (1)

#### 2.3.3. MDA Content Measurement

MDA content was determined according to the modified method of Avis et al. [27]. Approximately 0.5 g fresh mycelia were ground into powder in liquid nitrogen and homogenized with 5 mL of 10% (*w*/*v*) trichloroacetic acid (TCA). The homogenate was centrifuged at 8000 rpm for 15 min at 4 °C, and the supernatant was collected for subsequent assays. A 0.5 mL aliquot of supernatant was mixed with an equal volume of 0.6% thiobarbituric acid (TBA) solution (prepared with 10% TCA). The mixture was boiled for 15 min, rapidly cooled on ice, and centrifuged at 10,000 rpm for 10 min at room temperature. The absorbance of the supernatant was measured at 532 nm and 600 nm, denoted as A_532_ and A_600_, respectively.

MDA (nmol/g FW) = [(A_532_ − A_600_) × V]/(ε × d × W) (2)

where V is the total volume of extract (mL); ε is the molar extinction coefficient of the MDA-TBA complex (155 mM^−1^·cm^−1^); d is the cuvette optical path length (cm); and W is the fresh weight of the sample.

### 2.4. Transcriptome Sequencing Analysis Under Salt Stress

#### 2.4.1. Sample Preparation and Total RNA Extraction

Mycelia were collected after NaCl treatment at concentrations of 0, 1.0, 1.5, and 2.0 mol/L as described in Section 2.3.1, snap-frozen in liquid nitrogen, and total RNA was extracted using QIAzol Lysis Reagent (Qiagen, Hilden, Germany) according to the manufacturer’s instructions.

RNA concentration and purity were detected with a NanoDrop spectrophotometer (Thermo Fisher Scientific Inc., Waltham, USA), whereas RNA integrity was verified on an Agilent 2100 Bioanalyzer [28]. Qualified RNA samples satisfied the following thresholds: OD_260_/OD_280_ = 1.8–2.4, OD_260_/OD_230_ ≥ 1.5, and RIN ≥ 6.0 for fungi, and 28S/18S rRNA ratio ≥ 1.0. Only samples that satisfied all quality standards were used for cDNA library construction.

#### 2.4.2. Library Construction and Sequencing

cDNA libraries were enriched from qualified total RNA via oligo (dT) magnetic beads, fragmented, reverse-transcribed into double-stranded cDNA, and subjected to paired-end 150 bp sequencing on the Illumina NovaSeq X Plus platform (Illumina, San Diego, CA, USA) [29]. Raw sequencing reads were preprocessed using Trim Galore! (v0.6.7) with default parameters to remove low-quality bases (Phred < 20), ambiguous N bases, and Illumina adapter sequences, yielding clean reads. Global quality metrics including Q20/Q30 base quality, average read length, and GC content were calculated to evaluate sequencing reliability.

Clean reads were mapped to the *de novo* assembled genome of *A. westerdijkiae* strain NDX1 using HISAT2 (v2.1.0) with standard parameters [30]. The in-house generated GFF3 gene annotation file produced from the same genome assembly pipeline was applied for subsequent transcript quantification.

Raw read counts per gene were calculated by RSEM (v1.3.3) based on mapped clean reads [31], and gene expression levels were normalized to FPKM (Fragments Per Kilobase of transcript per Million mapped reads) values for inter-group expression comparison. The calculation formula of FPKM was defined as:

FPKM = total exon fragments/[mapped reads (millions) × exon length (kb)](3)

All raw RNA-seq fastq reads, assembled genome contigs and full gene annotation files have been deposited into the NCBI SRA. (accessions: SRR39720775, SRR39720776, SRR39720777, SRR39720778). All raw sequencing datasets will be released to the public synchronously upon the online publication of this manuscript.

#### 2.4.3. Functional Enrichment Analysis

Gene Ontology (GO) functional annotation and Kyoto Encyclopedia of Genes and Genomes (KEGG) pathway enrichment analysis were performed on high-confidence DEGs [32]. We prioritized functional enrichment of genes participating in the HOG-MAPK signaling, glycerol biosynthesis, ion transport, and plasma membrane homeostasis pathways.

### 2.5. Quantitative Real-Time PCR (qRT-PCR) Validation

A total of 12 salt stress-responsive DEGs were selected for qRT-PCR validation. Total RNA was extracted following the protocol in Section 2.3.1, and reverse-transcribed into cDNA using the PrimeScript™ RT Kit (TaKaRa, Dalian, China). Tubulin was used as the internal reference gene. qRT-PCR amplification was carried out on a Bio-Rad CFX96 Real-Time System (Bio-Rad, Hercules, CA, USA) with TB Green^®^ Premix Ex Taq™ II (TaKaRa, Dalian, China).

All primer sequences used in this study are listed in Supplementary Table S1. The 10 μL reaction system consisted of 5 μL TB Green^®^ Premix Ex Taq™ II, 0.4 μL forward primer (10 μmol/L), 0.4 μL reverse primer (10 μmol/L), 1 μL cDNA template, and 3.2 μL RNase-free ddH_2_O. The amplification program was set as: 95 °C pre-denaturation for 30 s; 40 cycles of 95 °C denaturation for 15 s, 62 °C annealing and extension for 30 s; followed by a melting curve analysis from 65 °C to 95 °C with 0.5 °C increments per 5 s to confirm the specificity of amplification products. Relative gene expression was calculated using the 2^−ΔΔCt^ algorithm [33] and converted to log_2_FC for cross-comparison with RNA-seq results.

### 2.6. Statistical Analysis

All physiological experiments (colony diameter, relative electrical conductivity, MDA content and qRT-PCR verification) were conducted with three independent biological replicates and three technical replicates for each treatment group. Data were expressed as mean ± standard deviation (SD). One-way analysis of variance (ANOVA) followed by Tukey’s multiple comparison test was performed using GraphPad Prism 11.0 to assess significant differences between different NaCl concentration groups, with the significance threshold set at *p* < 0.05.

RNA-seq raw data processing, DEG screening, GO/KEGG enrichment and correlation analysis were completed online using the Majorbio Cloud Platform (www.majorbio.com), a professional multi-omics data analysis cloud service. Genes were preliminarily defined as DEGs with padj < 0.001 and |log_2_(fold change, FC)| ≥ 1; transcripts satisfying |log_2_FC| ≥ 2 were further selected for functional enrichment. Pearson correlation analysis was performed to evaluate transcriptional covariation between Hog1 and its downstream target genes. Heatmaps, Venn diagrams and bubble enrichment plots were generated via the built-in visualization tools on the Majorbio Cloud Platform (https://cloud.majorbio.com/).

## 3. Results

### 3.1. Effects of Salt Stress on Growth Phenotype and Plasma Membrane Physiological Characteristics of A. westerdijkiae NDX1

To investigate the effects of salt stress on the growth and plasma membrane physiological characteristics of *A. westerdijkiae* NDX1, gradient NaCl treatments at 0, 1.0, 1.5, and 2.0 mol/L were established. Colony morphology, colony diameter, relative electrical conductivity (REC), and malondialdehyde (MDA) content were determined systematically.

**Figure 1:**
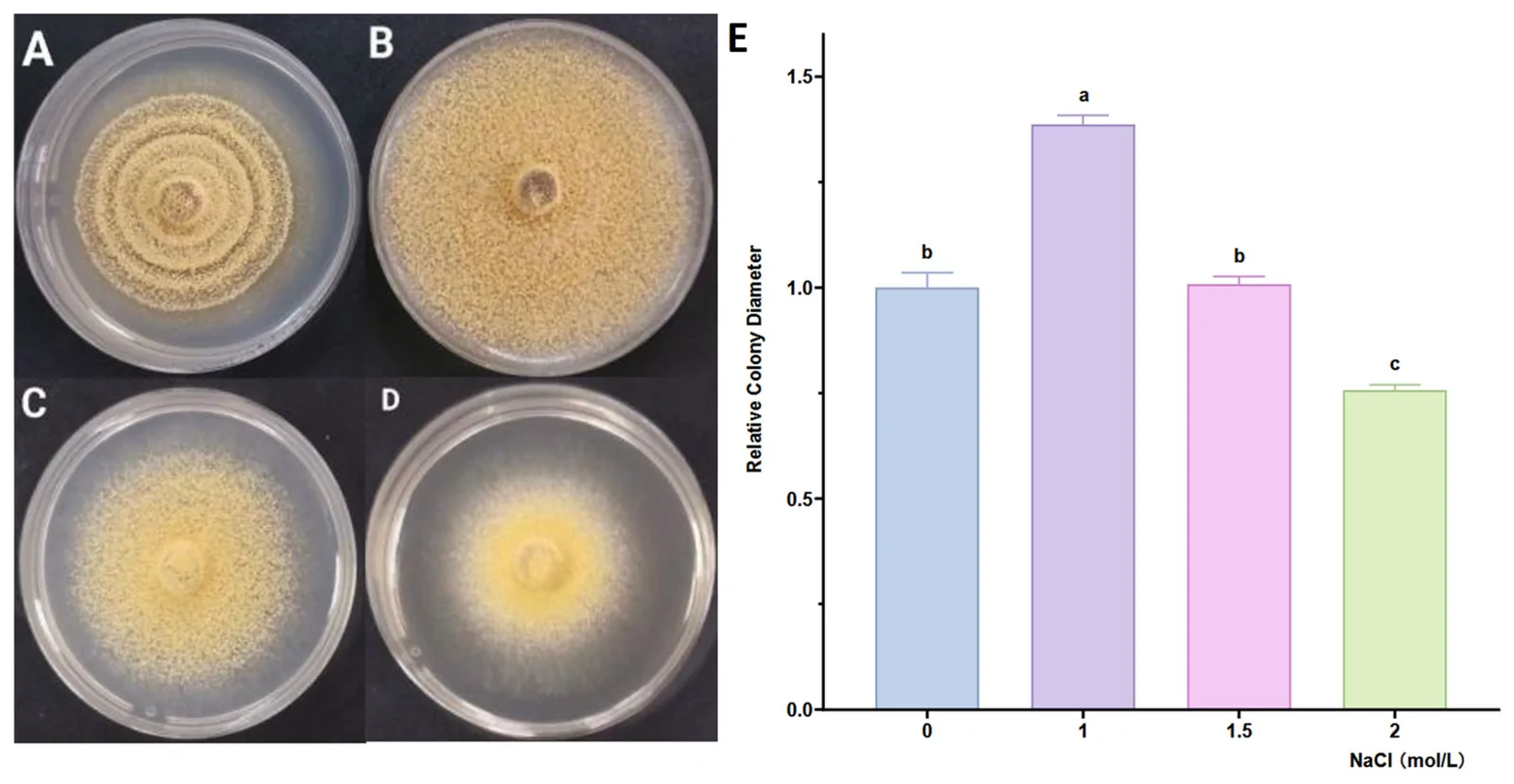
Colony morphology of *A. westerdijkiae* NDX1 under different NaCl concentrations. (**A**–**D**) represent colony morphology after 7 days of incubation under 0, 1.0, 1.5, and 2.0 mol/L NaCl, respectively; (**E**) shows the statistical results of colony diameter. Data are presented as the mean ± standard error (SE) of three independent biological replicates. Different lowercase letters indicate significant differences at *p* < 0.05.

**Figure 2:**
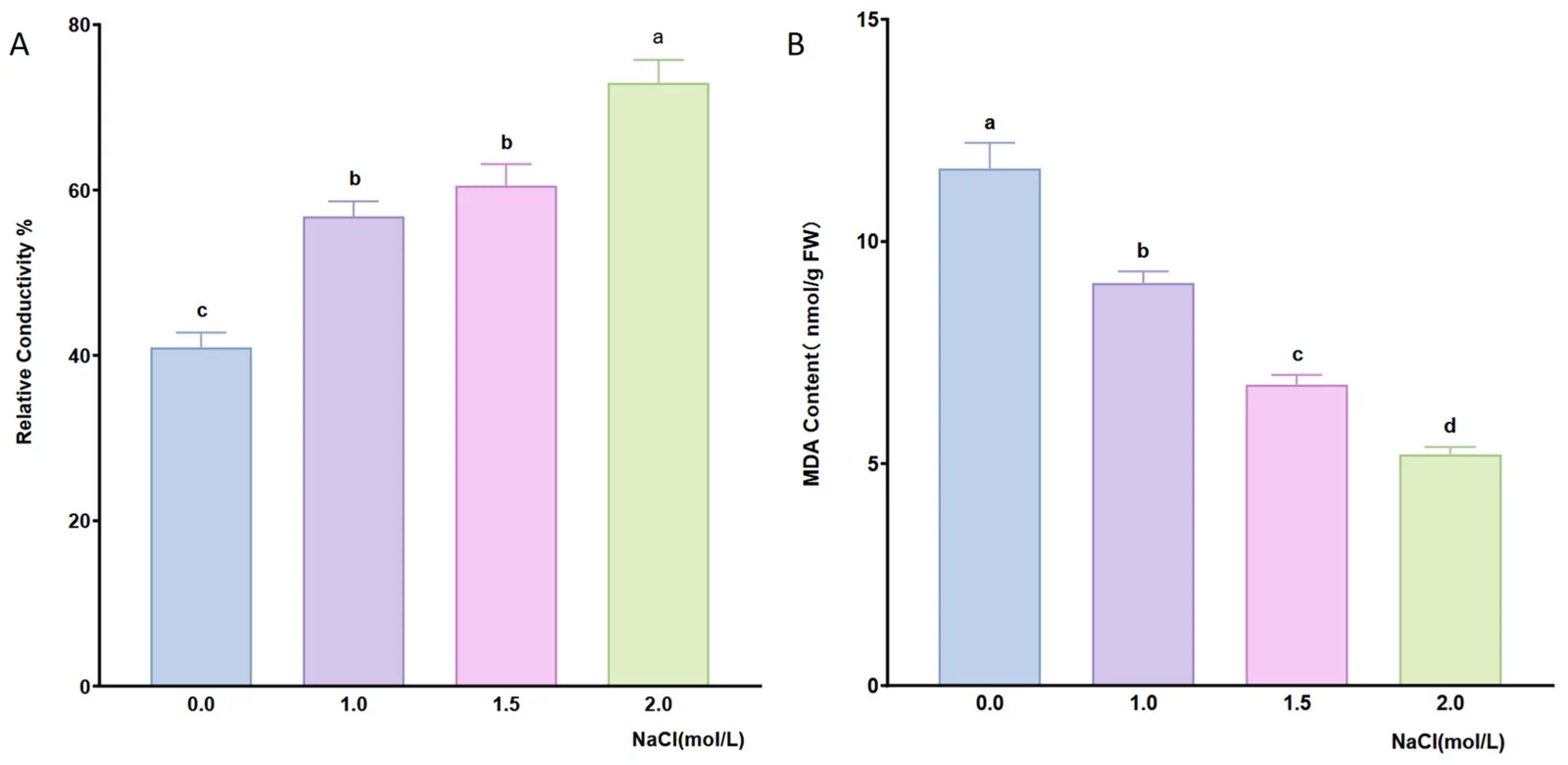
Effects of different NaCl concentrations on plasma membrane integrity and lipid peroxidation of *A. westerdijkiae* NDX1. (**A**,**B**) relative electrical conductivity (REC) and malondialdehyde (MDA) content. Mycelia were treated with 0, 1.0, 1.5, and 2.0 mol/L NaCl for 2 days before determination. Data are presented as the mean ± standard error (SE) of three independent biological replicates. Different lowercase letters above bars indicate significant differences at *p <* 0.05 by one-way ANOVA followed by Duncan’s multiple range test.

Salt stress markedly altered the colony morphological traits and vegetative growth rate of the strain. Under the 0 mol/L NaCl control, colonies grew in regular concentric rings with neat margins and dense conidial distribution. Treatment with 1.0 mol/L NaCl induced intensified pigment deposition and accelerated mycelial expansion, leading to approximately 35% larger colony diameter relative to the control. At 1.5 mol/L NaCl, no statistical difference in colony diameter was observed relative to the control, whereas colony color exhibited obvious fading, conidia became sparsely arranged, and the typical concentric ring structure disappeared. When the NaCl concentration increased to 2.0 mol/L, the colony color further lightened, conidial distribution became sparser, and the colony diameter decreased by more than 25%, indicating a pronounced inhibitory effect of high salinity on mycelial growth (Figure 1). REC value increased progressively alongside elevated NaCl concentration. Compared with the control group, REC increased by about 15% in the 1.0 and 1.5 mol/L treatments, and reached an increase of nearly 30% at 2.0 mol/L NaCl, showing extremely significant differences among treatments. By contrast, MDA content decreased progressively with rising salinity and dropped to the lowest level at 2.0 mol/L NaCl across the entire concentration gradient (Figure 2).

The overall trend indicated that low-salt stimulation and high-inhibition growth phenotypes; mild salinity promoted mycelial proliferation, moderate salinity modified colony morphology without obvious biomass loss, whereas hypersalinity suppressed vegetative development. Meanwhile, REC was positively correlated with NaCl concentration, whereas MDA content was negatively correlated with salinity, presenting an opposite variation pattern between the two physiological indices.

### 3.2. Overview of Transcriptome Sequencing Data Under Salt Stress

Transcriptome sequencing was performed on mycelial samples treated with 0, 1.0, 1.5, and 2.0 mol/L NaCl. A total of 46.2, 46.8, 53.5, and 55.1 million raw reads were generated from the four groups, respectively. After quality control, low-quality reads and adapter sequences were removed to generate high-quality clean reads. The uniquely mapped efficiencies to the reference genome ranged from 93.86% to 95.26%, and the number of effectively annotated expressed genes was between 9719 and 9916 (Table 1).

Overall, the transcriptome data presented high mapping efficiency, uniform genome coverage, and complete gene annotation, indicating reliable sequencing quality. The sequencing data were qualified for subsequent differential gene screening, functional enrichment, and pathway mechanism analysis.

### 3.3. Analysis of Differentially Expressed Genes (DEGs) Under Salt Stress

#### 3.3.1. Statistical Analysis of DEG Quantity

**Figure 3:**
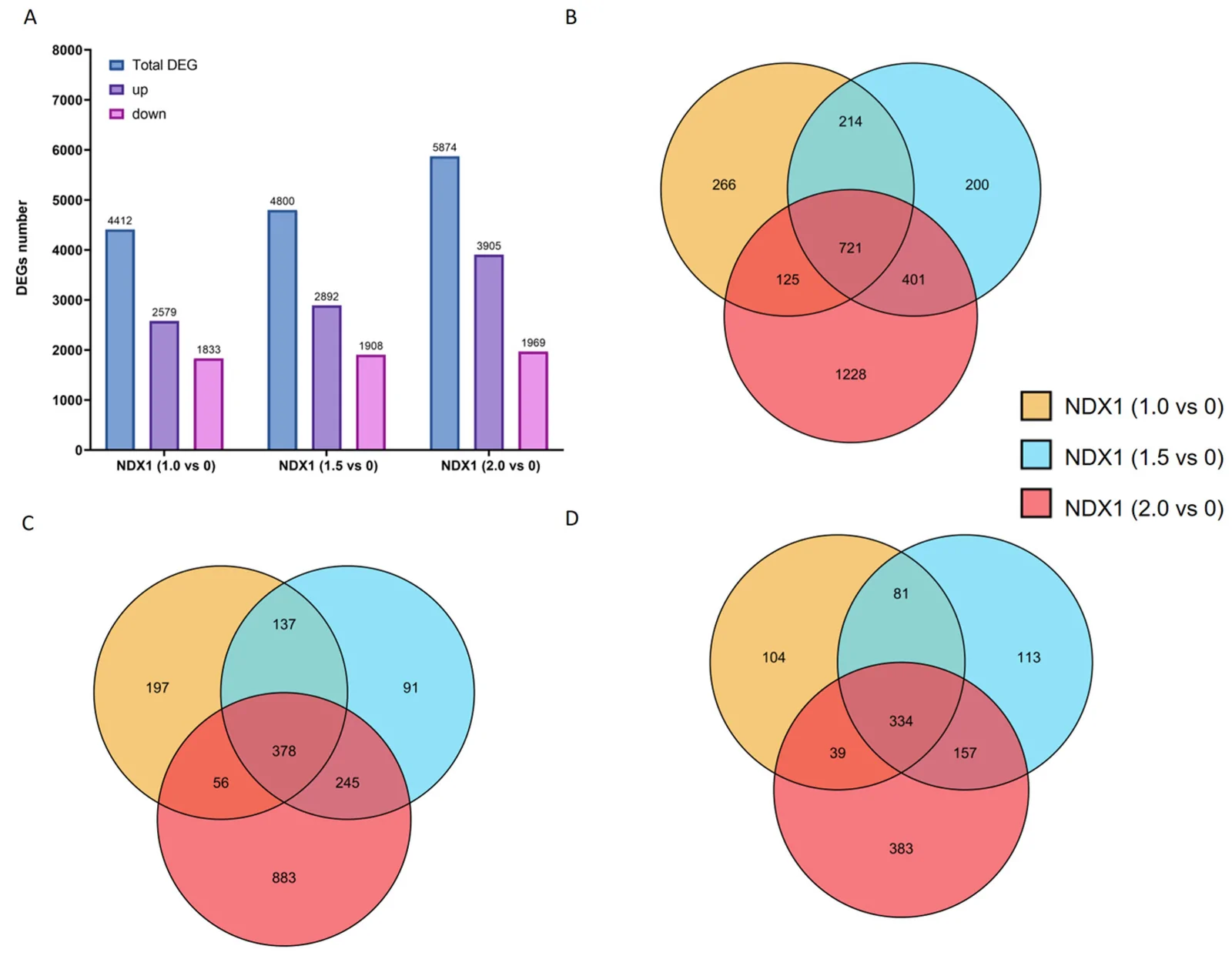
Statistical analysis of DEGs under different NaCl concentrations. (**A**) quantity statistics of DEGs in each treatment group; (**B**–**D**) Venn diagram of all DEGs, upregulated and downregulated DEGs, respectively. 1.0 vs. 0, 1.5 vs. 0, and 2.0 vs. 0 represent pairwise comparisons of 1.0, 1.5, and 2.0 mol/L NaCl treatments versus the 0 mol/L control group, respectively. All DEGs were initially screened under the thresholds of padj < 0.001 and |log_2_FC| ≥ 1, and 3155 high-confidence DEGs with |log_2_FC| ≥ 2 were further selected for subsequent functional analysis.

DEGs were screened based on the thresholds of padj < 0.001 and |log_2_FC| ≥ 1, and a total of 6976 DEGs were identified (Figure 3A). To obtain high-confidence genes for GO and KEGG enrichment, we further filtered transcripts with |log_2_FC| ≥ 2, and a total of 3155 high-confidence DEGs were retained for subsequent functional characterization. Based on this rigorous screening strategy, we further statistically characterized the DEG distribution at different salt concentrations. 

Compared with the 0 mol/L control group, 1326 (768 upregulated, 558 downregulated), 1536 (851 upregulated, 685 downregulated), and 2475 DEGs (1562 upregulated, 913 downregulated) were identified under 1.0, 1.5, and 2.0 mol/L NaCl, respectively (Figure 3B–D). The total number of DEGs, as well as the amounts of upregulated and downregulated genes, increased gradually with the elevation of salt concentration (Figure 3). At all tested salinity gradients, significantly more genes were upregulated than downregulated, and the numerical difference between up- and downregulated DEGs expanded with stronger osmotic stress. This trend preliminarily suggests that the fungus mobilizes an expanding repertoire of functional genes to counteract escalating osmotic injury.

Venn diagram analysis (Figure 3) further demonstrated divergent transcriptional signatures across salinity treatments: the transcriptomic profile of the 1.0 mol/L NaCl group was clearly distinct from both the 0 mol/L control and the 2.0 mol/L high-salt group. A total of 266 DEGs (197 upregulated, 104 downregulated) were uniquely detected under mild 1.0 mol/L NaCl, with no corresponding expression changes observed at 1.5 and 2.0 mol/L NaCl.

And Venn analysis also revealed 721 cores shared DEGs across all three salt-stressed groups, including 378 co-upregulated and 334 co-downregulated genes (Figure 3B). The existence of this set of universal salt-responsive genes revealed a conserved core transcriptional regulatory module for osmotic adaptation in *A. westerdijkiae*. Meanwhile, the gradual expansion of DEG pools at higher salinity confirmed that the strain’s transcriptional response magnitude is positively correlated with salt stress intensity, and more functional pathways are mobilized to cope with severe hyperosmotic pressure.

#### 3.3.2. GO and KEGG Enrichment Analysis

Gene Ontology (GO) annotation and Kyoto Encyclopedia of Genes and Genomes (KEGG) pathway enrichment analysis were performed on all DEGs.

**Figure 4:**
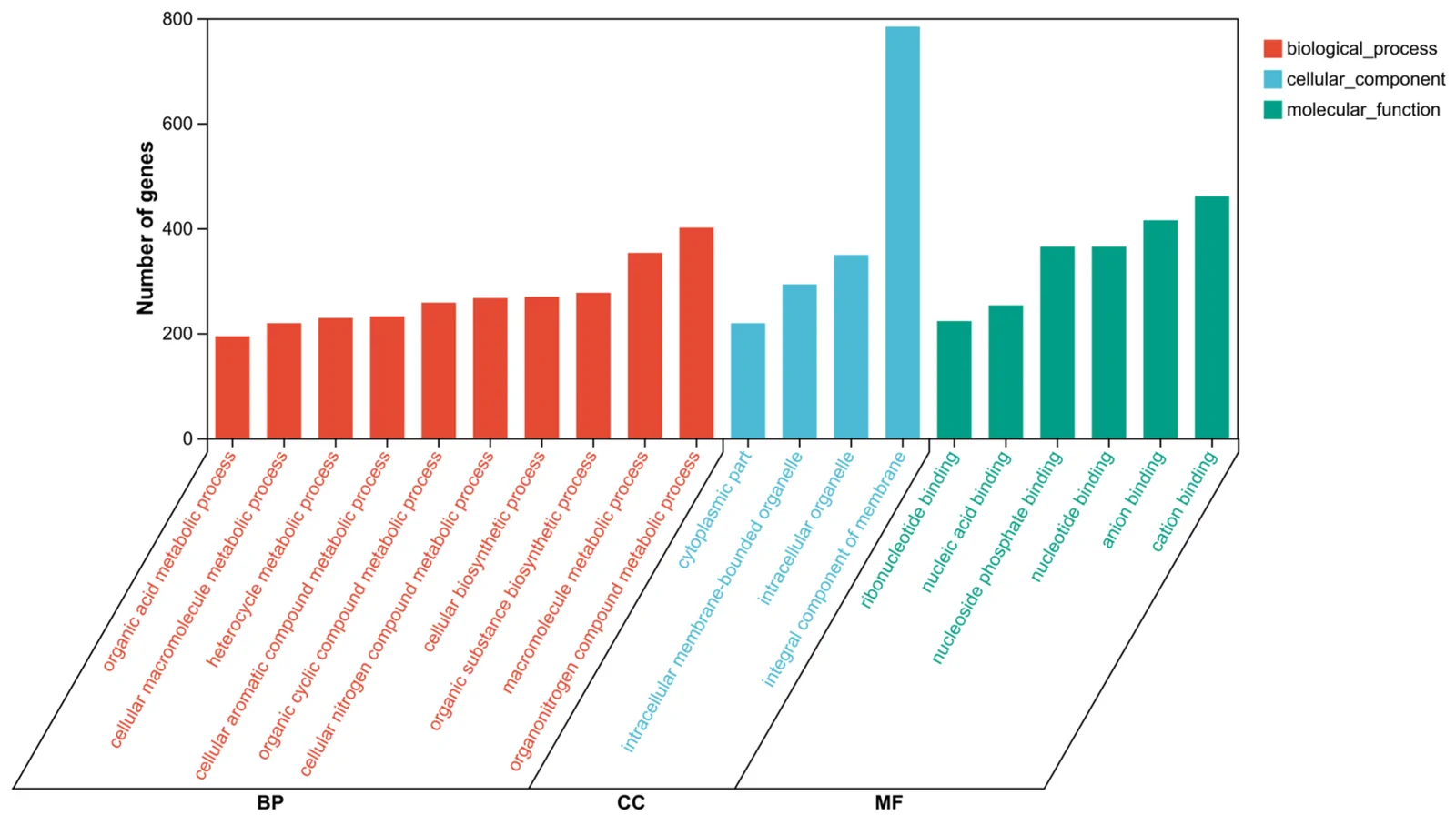
GO functional annotation of DEGs of *A. westerdijkiae* NDX1 under salt stress.

GO results showed that in the biological process category, DEGs were mainly enriched in fundamental metabolic processes, including organic acid metabolism, macromolecule metabolism, and nitrogen compound metabolism. In the cellular component category, DEGs were primarily annotated to plasma membrane components and intracellular membrane-bounded organelles. In the molecular function category, cation binding, nucleic acid binding, and nucleotide binding were the dominant functional terms (Figure 4).

**Figure 5:**
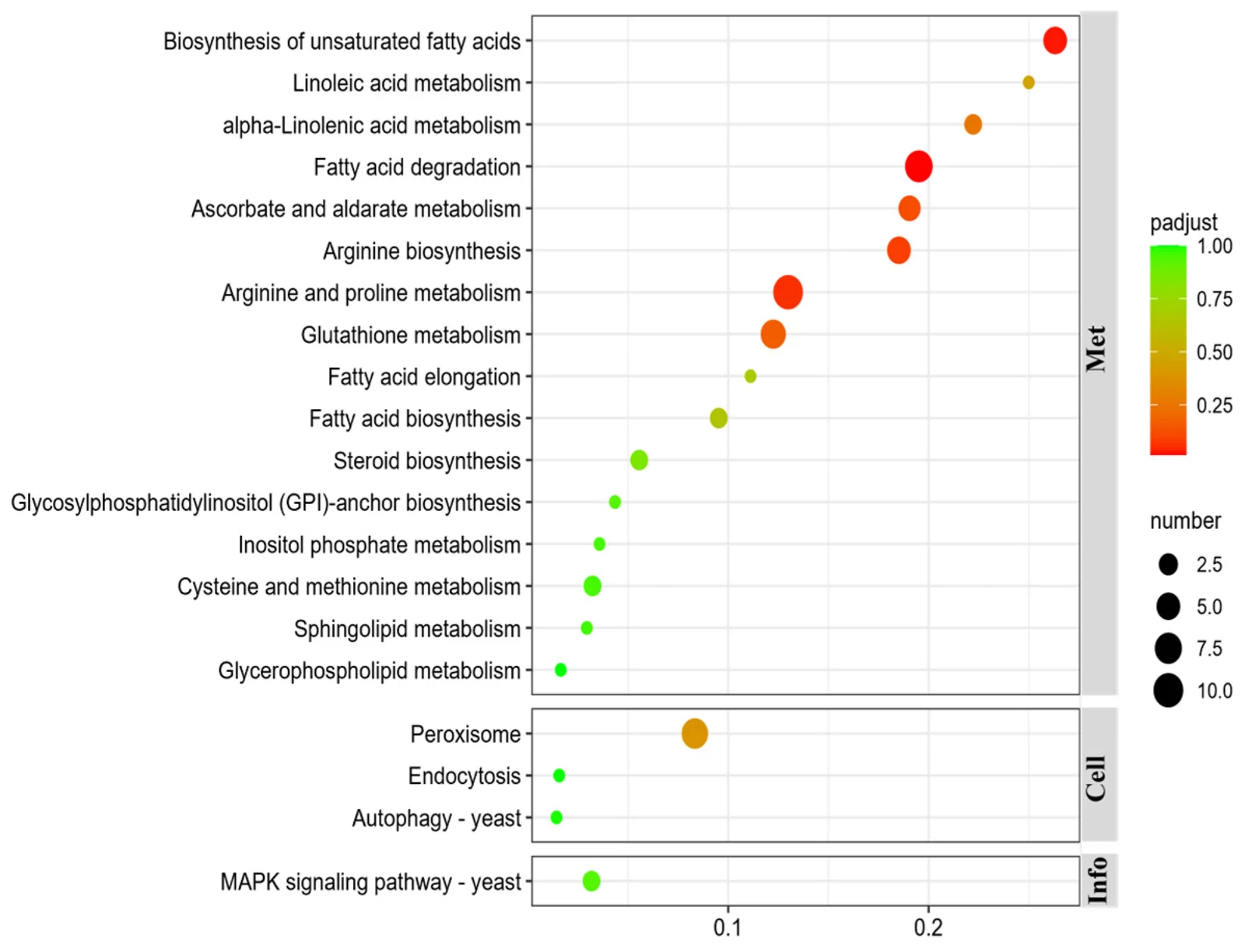
KEGG pathway enrichment of DEGs of *A. westerdijkiae* NDX1 under salt stress.

KEGG enrichment analysis demonstrated that DEGs were mainly assigned to three major functional categories: substance metabolism, cellular processes, and signal transduction. The most significantly enriched pathways included unsaturated fatty acid biosynthesis, fatty acid degradation, arginine/proline metabolism, glutathione metabolism, the yeast MAPK signaling pathway, peroxisomes, and autophagy (Figure 5). A number of DEGs annotated as polyketide synthases (PKS), nonribosomal peptide synthetases and other secondary metabolic proteins were also identified across the NaCl concentration gradients (Table S2).

GO functional classification of the complete 266 salinity-specific DEGs was performed to characterize their overall functional profiles (Supplementary Figure S1). The GO results revealed prominent enrichment in membrane structural components, ion binding activity, and organic nitrogen/macromolecule metabolic processes, indicating comprehensive remodeling of the cell membrane and primary metabolism under mild salinity.

**Figure 6:**
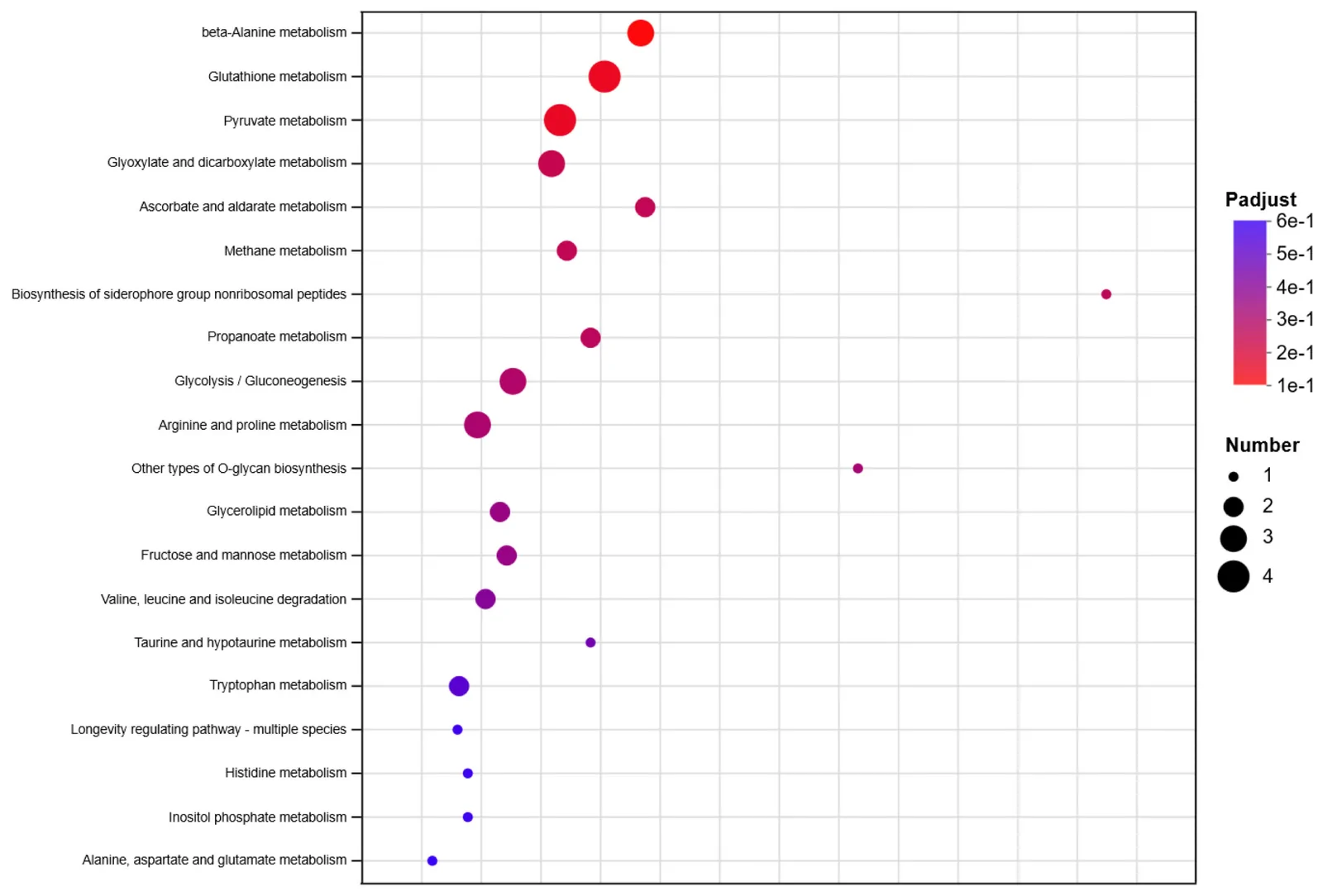
KEGG pathway enrichment of 197 DEGs of *A. westerdijkiae* NDX1 under 1 mol/L NaCl salt stress.

To further dissect the growth-promoting metabolic program exclusive to low salinity, KEGG enrichment was separately conducted on the 197 specifically upregulated DEGs, with the core enrichment results shown in Figure 6. These induced genes were significantly enriched in glycolysis, amino acid biosynthesis, glycerolipid metabolism and glutathione antioxidant pathways, which coordinately supply energy, compatible osmolytes and membrane lipids to support rapid mycelial expansion.

In contrast, KEGG enrichment of the 104 low-salt-specific downregulated DEGs (Supplementary Figure S2) showed suppressed catabolic and damage-repair pathways, including fatty acid degradation, lysine breakdown and nucleotide excision repair. Transcriptional repression of these energy-consuming pathways allows the strain to redirect metabolic resources toward vegetative growth rather than severe stress defense.

#### 3.3.3. Expression Characteristics of Key Genes Under Salt Stress

**Figure 7:**
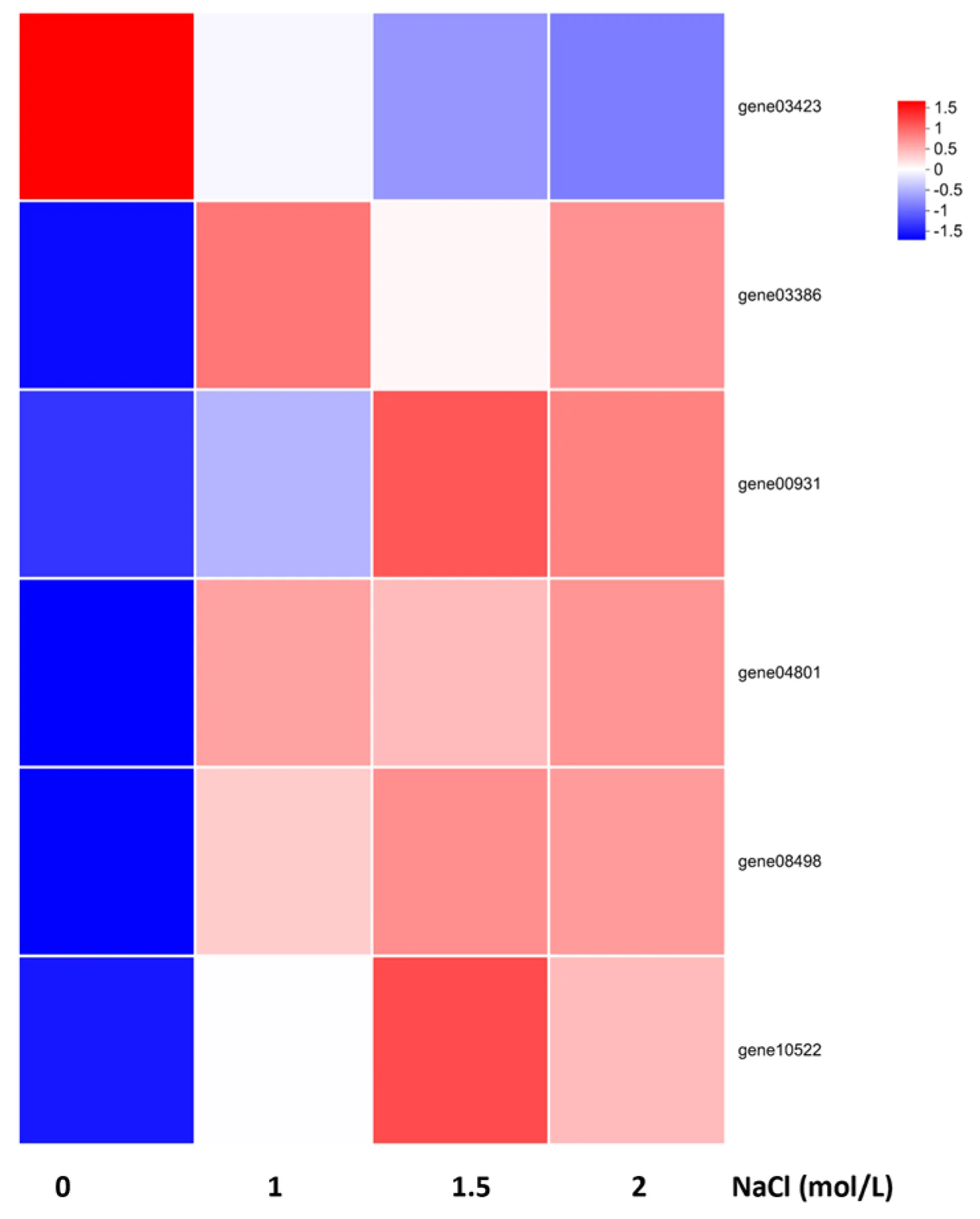
Heat map of key REC-related gene expression in *A. westerdijkiae* NDX1 under salt stress.

Six key genes involved in ion transport, proton pump synthesis, and plasma membrane homeostasis were screened from the transcriptome DEGs (Table 2). Gene expression heat maps revealed distinct expression patterns under gradient NaCl treatments. Among these genes, five candidates were progressively upregulated with rising NaCl dosage, whereas gene03423 was persistently suppressed (Figure 7).

Gene IDs are listed on the right. The color scale represents normalized gene expression (log_2_-transformed fold change), with red indicating upregulation and blue indicating downregulation. Columns correspond to 0, 1.0, 1.5, and 2.0 mol/L NaCl treatments, respectively.

**Figure 8:**
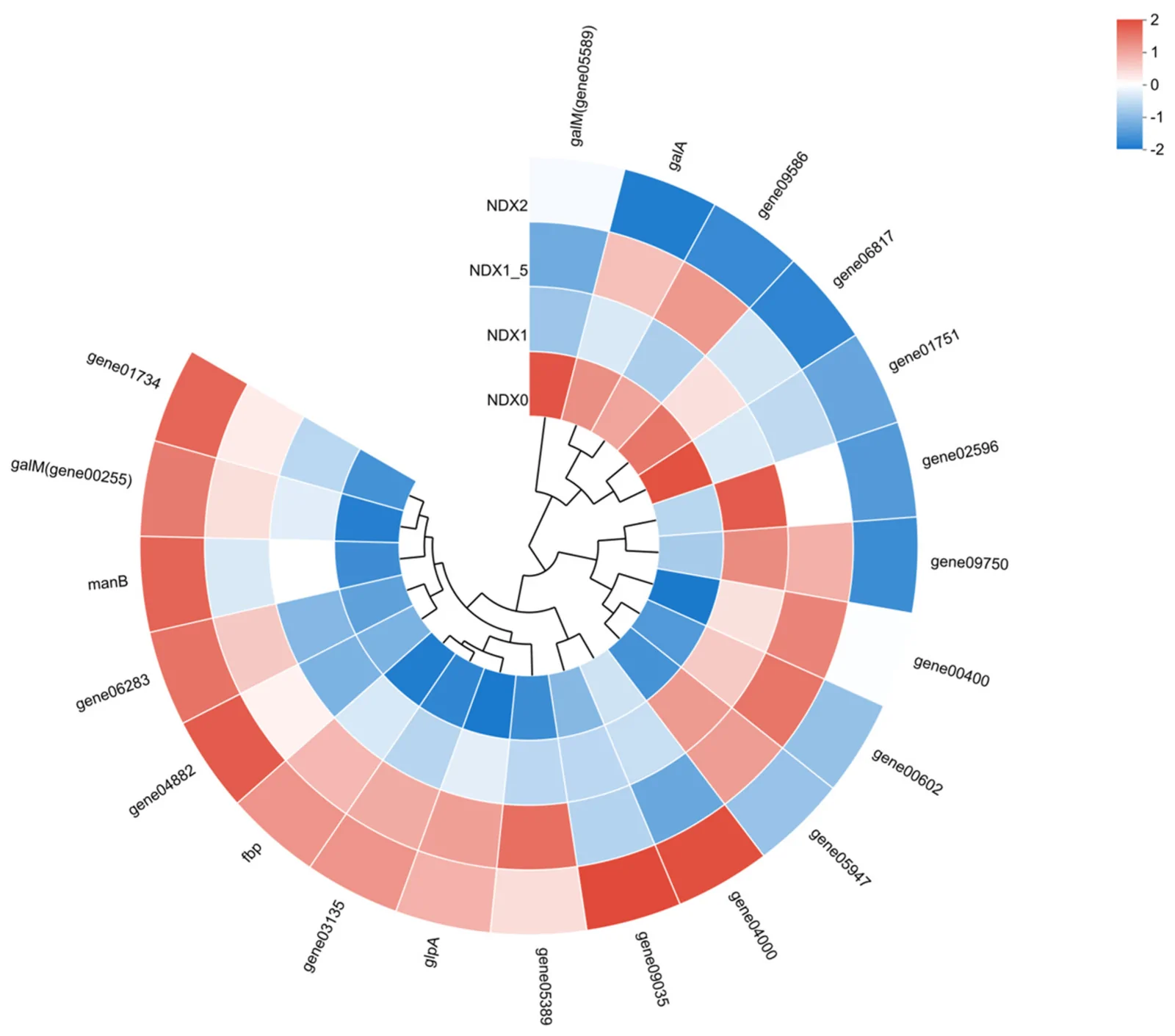
Circular heatmap of glycerol metabolism-related key gene expression in *A. westerdijkiae* NDX1 under salt stress.

A total of 21 key genes associated with glycerol biosynthesis, carbon precursor supply, lipid recycling, and glycerol transmembrane transport were also identified (Table 3). Collectively, genes governing *de novo* glycerol biosynthesis exhibited dose-dependent transcriptional induction, with the highest expression levels observed under 2.0 mol/L NaCl. Genes participating in glycolysis, carbohydrate metabolism, lipid catabolism, and glycerol transport were all significantly induced under salt stress (Figure 8).

**Figure 9:**
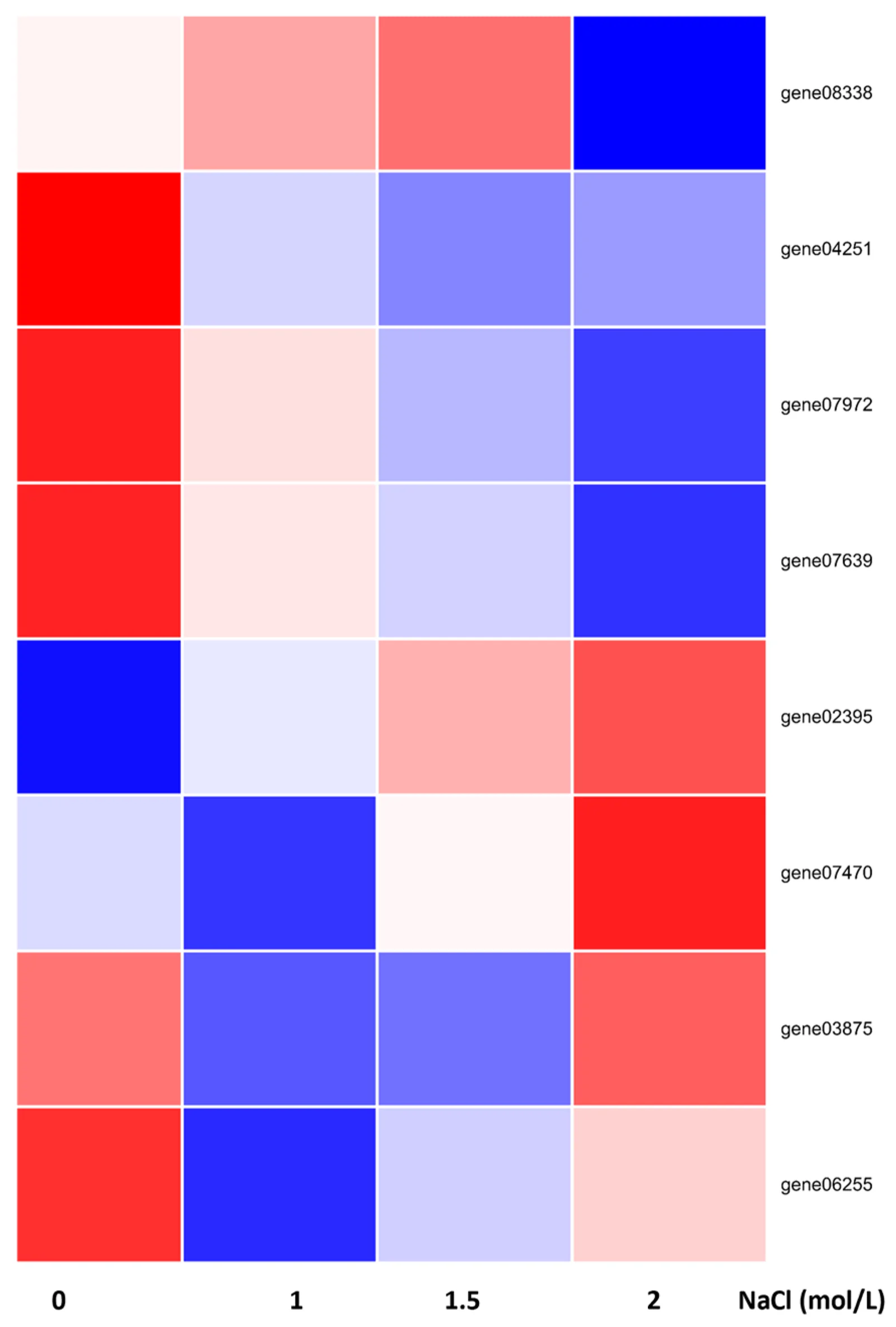
Heat map of the HOG-MAPK pathway-related key gene expression in *A. westerdijkiae* NDX1 under salt stress.

A total of eight key differentially expressed genes belonging to the HOG-MAPK signaling pathway were identified, covering the whole processes of osmotic perception, upstream kinase cascade reaction, and terminal kinase response (Table 4). Heat map analysis showed that pathway members exhibited concentration-dependent expression changes under salt stress. The expression patterns of osmotic sensor genes, upstream MAPKKK, and midstream MAPKK differed among treatment groups, while two terminal *Hog*1-coding genes were significantly and persistently upregulated alongside salinity elevation (Figure 9).

Pearson correlation analysis was performed between the key kinase Hog1 encoding gene (gene02395) and downstream functional genes involved in ion transport, glycerol synthesis and ATP synthesis (Table 5). The results showed that *Hog*1 exhibited a perfect positive correlation trend with gene08498 and gene03135 (*r* = 1.000, *p* = 0.083), and a perfect negative correlation trend with gene03423 (*r* = −1.000, *p* = 0.083). Strong positive correlation trends (*r* = 0.800) were also observed between *Hog*1 and gene10522, gene09494, gene00931 and gene04801. Despite *p* > 0.05 for all correlation indices, these transcripts displayed coordinated transcriptional covariation, providing transcriptional clues for the potential downstream regulatory association of the HOG-MAPK pathway.

### 3.4. Validation of Transcriptome Data by qRT-PCR

Twelve salt-responsive differentially expressed genes were randomly selected to verify the reliability of RNA-seq data via qRT-PCR. Overall, the expression fold-change trends of all tested genes were highly consistent between qRT-PCR and transcriptome datasets (Figure S3). Pearson correlation analysis further revealed strong positive correlations between the two detection platforms: the correlation coefficients reached 0.9720 (*p* < 0.001, 1.0 mol/L vs. control), 0.7767 (*p* = 0.0030, 1.5 mol/L vs. control), 0.7297 (*p* = 0.0071, 2.0 mol/L vs. control), and 0.8620 (*p* < 0.001) for all combined samples (Figure S4). The highly matching transcriptional profiles and significant cross-platform correlation collectively demonstrated the authenticity and credibility of the RNA-seq DEG data. These results could be used for further exploration of the molecular mechanism underlying salt tolerance in this strain.

## 4. Discussion

In the present research, we systematically deciphered the salt-adaptive traits of NDX1 at the phenotypic, physiological, and transcriptomic levels, establishing a multi-layered regulatory network consisting of vegetative adaptation, membrane-ion homeostasis, glycerol metabolic reprogramming, and HOG-MAPK-mediated signal transduction. These findings provide fundamental data and candidate target genes for risk control of toxigenic halotolerant *Aspergillus* in fermented foods.

### 4.1. Concentration-Dependent Growth and Membrane Physiological Adaptation of NDX1 Under Salt Stress

This study confirmed that *A. westerdijkiae* NDX1 exhibits a typical low-promotion and high-inhibition growth pattern in response to salt concentration [6,24]. Low salinity significantly promoted mycelial expansion, while high salinity markedly inhibited colony growth. This phenomenon has been widely reported in salt-tolerant species such as *A. sydowii* and *A. glaucus*, and is regarded as a common ecological adaptive feature of food-derived salt-tolerant fungi [1,5,24]. A recent multi-omics study on halophilic *A. sydowii* also confirmed that fungal physiological responses to salinity do not follow a simple graded trend with increasing salt concentration, and distinct adaptive strategies are activated under optimal salinity and hypersaline stress [34]. Low-level salinity may mildly activate basal energy metabolism and nutrient transport without inducing severe osmotic injury, thus accelerating mycelial proliferation, consistent with the hormesis effect of moderate abiotic stress on filamentous fungi [35]. Mild salinity pre-activates the osmoprotection and energy metabolic pathways to facilitate mycelial expansion. In contrast, high salinity causes excessive intracellular Na^+^ accumulation, disrupting ion balance and plasma membrane integrity. The strain reduces ion uptake pressure by restricting mycelial expansion, which conforms to the volume compensation adaptive strategy commonly found in salt-tolerant fungi [24]. Even under 2.0 mol/L high-salt treatment, the strain still maintained considerable growth capacity, indicating its strong adaptability to high-salt fermented habitats [1].

As summarized in DEG quantitative statistics (Figure 3A), significantly more transcripts were upregulated than downregulated across all salt gradients. This numerical imbalance reflects the core adaptive logic of this strain: salt stress triggers large-scale transcriptional activation of anabolic, ion transport and osmoprotective pathways to counteract osmotic pressure, while only a small subset of energy-consuming redundant stress catabolic modules is repressed to conserve cellular resources. Such global transcriptional activation is consistent with the extensive metabolic remodeling mode reported in halotolerant *Aspergillus* strains [36].

The transcriptomic data further uncovered the molecular basis of this bidirectional salinity-dependent growth phenotype. Mild 1.0 mol/L NaCl activated anabolic and energy-supplying pathways that drive rapid mycelial proliferation, matching the metabolic remodeling profiles of salt-tolerant *Aspergillus* under optimal low salinity [36]. Genes involved in the TCA cycle, mitochondrial ATP synthesis and amino acid transmembrane transport were markedly upregulated at 1.0 mol/L NaCl, while this transcriptional activation was largely abolished under 2.0 mol/L high salinity. Transcripts uniquely induced only at moderate salinity form an independent transcriptional signature that separates the 1.0 mol/L group from both the control and high-salt treatments.

Consistent with the non-linear transcriptional divergence among treatment groups shown in transcriptome profiling, this mild-salt-exclusive gene pool is the core driver of enhanced vegetative activity under 1.0 mol/L NaCl. Integrated functional pathway analysis of these salinity-specific transcripts reveals a coordinated metabolic trade-off: the fungus simultaneously upregulates carbon metabolism, energy generation and membrane remodeling programs to supply precursors and ATP for hyphal growth, while repressing high-cost antioxidant detoxification pathways to divert more resources toward proliferation.

By comparison, high salinity forced the fungus to allocate most cellular resources to osmotic defense systems including the HOG signaling cascade and glycerol biosynthesis [10,36], thereby repressing growth-associated metabolic programs and ultimately resulting in suppressed vegetative growth, which matches the energy trade-off model of osmotic adaptation in filamentous fungi [24].

Plasma membrane integrity and lipid peroxidation levels are key physiological indices for evaluating salt-induced damage in microorganisms [27]. In the present study, relative electrical conductivity (REC) increased continuously with rising salinity, which is consistent with the established model that high salinity disrupts the phospholipid bilayer structure, impairs membrane protein function, and increases plasma membrane permeability, thereby triggering intracellular electrolyte leakage [3,4,25]. These physiological responses were consistent with those observed in extremely salt-tolerant yeasts and other *Aspergillus* species [4,25].

MDA is a classic indicator for evaluating the degree of lipid peroxidation, and lipid peroxidation is closely associated with stress-induced damage in fungal cells [27]. The decreased MDA levels observed in this experiment indicate a reduction in the extent of cellular lipid peroxidation in strain NDX1. Notably, NDX1 exhibited a unique physiological phenotype featuring elevated membrane permeability coupled with suppressed lipid peroxidation. This response pattern differed from that of most plants and salt-sensitive microorganisms, but was highly consistent with A. *sydowii* [24,36].

We inferred that the strain adopts a trade-off adaptation strategy: instead of rigidly maintaining membrane tightness, it remodels lipid metabolism to inhibit peroxidation reactions, reducing oxidative injury even with partial membrane damage [4,20]. Enhanced intracellular lipid catabolism supplies glycerol carbon precursors while consuming peroxidation-prone polyunsaturated fatty acids, which jointly leads to reduced MDA accumulation under hypersaline environments [20].

### 4.2. Transcriptional Regulation Mechanism of Plasma Membrane Homeostasis and Ion Transport Under Salt Stress

Upregulation of V-ATPase, mitochondrial ATP synthase, and high-affinity ion transporter genes indicated enhanced proton pumping to fuel active ion transmembrane transport for intracellular K^+^/Na^+^ rebalance. This indicates that the strain enhances proton pump activity and ATP synthesis to provide sufficient energy for active ion transmembrane transport. Meanwhile, high-affinity potassium and magnesium transport systems were activated to reconstruct intracellular K^+^/Na^+^ homeostasis and alleviate sodium ion toxicity [25].

Genes encoding low-affinity potassium transporters were markedly downregulated under high salt, implying that the strain can precisely switch potassium uptake modes according to ambient salt–potassium conditions. It shuts down inefficient low-affinity pathways and preferentially activates high-affinity systems, improving ion utilization efficiency and reducing energy consumption [25]. These results support previous findings that upregulation of ATP binding and proton pump activity serves as a pivotal key node for maintaining membrane potential and ion homeostasis [24]. The present study further demonstrated that NDX1 achieves membrane damage compensation and intracellular ion homeostasis through a coordinated mode of enhanced energy supply–remodeled ion transport–preferential activation of high-efficiency transport systems.

### 4.3. Osmotic Adaptation Mechanism Mediated by Glycerol Synthesis and Lipid Recycling

Glycerol is the primary compatible solute employed by filamentous fungi to cope with hyperosmotic stress [3,11,13]. A total of 21 key genes involved in glycerol synthesis, precursor supply, lipid recycling, and transmembrane transport were identified in this study, confirming that the strain remodels its osmotic metabolic network through coordinated regulation of multiple pathways. Rate-limiting genes such as glycerol-3-phosphate dehydrogenase were upregulated in a salt concentration-dependent manner, indicating significant activation of the *de novo* glycerol synthesis pathway. Synchronously expressed glycolysis and carbohydrate metabolism genes continuously provide carbon skeletons for glycerol production [11,13,19].

An important novel finding of this study is the significant upregulation of numerous lipid catabolism-related genes under salt stress. Transcriptomic data imply a potential involvement of lipid catabolic processes in supplying carbon precursors for glycerol synthesis under salt stress. Under carbon-limited conditions, intracellular triglycerides and phospholipids are degraded to provide endogenous synthetic substrates, forming a dual supply mode of exogenous carbon plus endogenous lipid resources [20,36]. On the one hand, this metabolic reprogramming ensures intracellular glycerol accumulation and osmotic balance maintenance. On the other hand, it remodels membrane lipid composition and inhibits lipid peroxidation at the source, reasonably explaining the decreased MDA content under high salinity [20,34]. Meanwhile, the coordinated upregulation of glycerol transport genes facilitates the synergy of synthesis and efflux, enabling precise regulation of intracellular and extracellular osmotic balance. Although direct metabolite quantification is absent, coordinated upregulation of the full set of lipid degradation and glycerol synthetic genes strongly supports the existence of this supplementary precursor pathway.

### 4.4. HOG-MAPK Signaling Pathway: A Key Regulatory Hub for Salt Tolerance

The HOG-MAPK pathway is a conserved key central cascade that enables fungi to sense osmotic stress and initiate downstream adaptive responses [3,10]. In this study, a complete gene cascade covering osmotic sensors, MAPKKK, MAPKK, and the terminal *Hog*1 was identified, constructing an intact osmotic signal transduction chain in *A. westerdijkiae*. The continuous upregulation of the terminal *Hog*1 transcripts with increasing salt concentration represented the key node mediating concentration-dependent pathway activation.

Previous studies on *A. nidulans* have confirmed that the HOG pathway mainly relies on the two-component system for upstream signal transduction [10]. In the current study, the expression patterns of upstream kinases exhibited interspecific divergence, whereas the *Hog*1-mediated downstream regulatory module remained highly conserved. Correlation analysis showed that *Hog*1 displayed obvious positive marginal and strong correlation trends with genes related to glycerol synthesis, ion transport, and energy metabolism, and presented a negative trend with low-affinity potassium transporters, forming a clear co-expression network at the transcriptional level. Despite all correlations, *p* > 0.05 due to the limited sample size; these coordinated transcriptional patterns possess clear biological implications. The non-significant *p* values restrict robust statistical proof of direct transcriptional regulation; the coordinated expression trend only provides suggestive transcriptional evidence rather than definitive regulatory causation.

Although *A. westerdijkiae* is well documented as an ochratoxin A producer in fermented food matrices [1,5], the present transcriptomic study was designed to unravel osmotic adaptive mechanisms instead of focusing on secondary metabolic pathways. Even so, the HOG-MAPK centered transcriptional regulatory model constructed in this work offers a fundamental reference for future research exploring the mechanistic crosstalk between salinity stress and mycotoxin biosynthesis [6,7].

### 4.5. Limitations and Ecological Considerations

While this work delineates the salt-adaptive regulatory network of *A. westerdijkiae* through physiological phenotyping and transcriptome profiling, several inherent experimental and culture-related limitations should be acknowledged for objective interpretation of the present findings.

Firstly, only discrete single-time-point salt treatments were set without continuous dynamic sampling, which weakens the statistical reliability of Hog1-gene correlations. All mechanistic conclusions rely solely on transcriptomic data; metabolomic and proteomic data are required to verify glycerol and lipid metabolic reprogramming.

Secondly, the proposed regulatory model is inferred merely from co-expression trends. Gene knockout/overexpression assays are needed to confirm the causal regulatory function of core HOG-MAPK genes [8]. All transcriptional covariations only reflect correlation rather than definite causation.

Thirdly, all cultures adopted shaking aeration conditions, while parallel aeration gradient comparisons were not performed. Though static low-oxygen pickling environments differ from our system, the oxygen-independent nature of the HOG-MAPK cascade and consistent changing trends of REC/MDA guarantee the qualitative credibility of our core findings. Static simulated fermentation can be adopted in follow-up research.

Fourthly, this work focuses on osmotic adaptation rather than secondary metabolism, and no gradient OTA detection was conducted, so we cannot establish quantitative links between salinity and ochratoxin accumulation.

### 4.6. Proposed Multi-Layer Salt-Adaptive Regulatory Model

As the central regulatory hub of osmotic signal transduction, Hog1 kinase coordinates three core adaptive programs at the transcriptional level: it upregulates genes governing glycerol synthesis, energy generation and high-affinity ion transport, while repressing transcripts encoding low-efficiency potassium transporters to maintain intracellular ion balance and energy homeostasis. This conserved regulatory mode is consistent with previous multi-omics reports on halotolerant *A. sydowii*, reflecting evolutionary conservation of the HOG-MAPK cascade across food-borne aspergilli [9]. The unique transcriptional signature of NDX1 also represents habitat-specific adaptive evolution under the high-salt, low-potassium microenvironment of fermented pickles.

**Figure 10:**
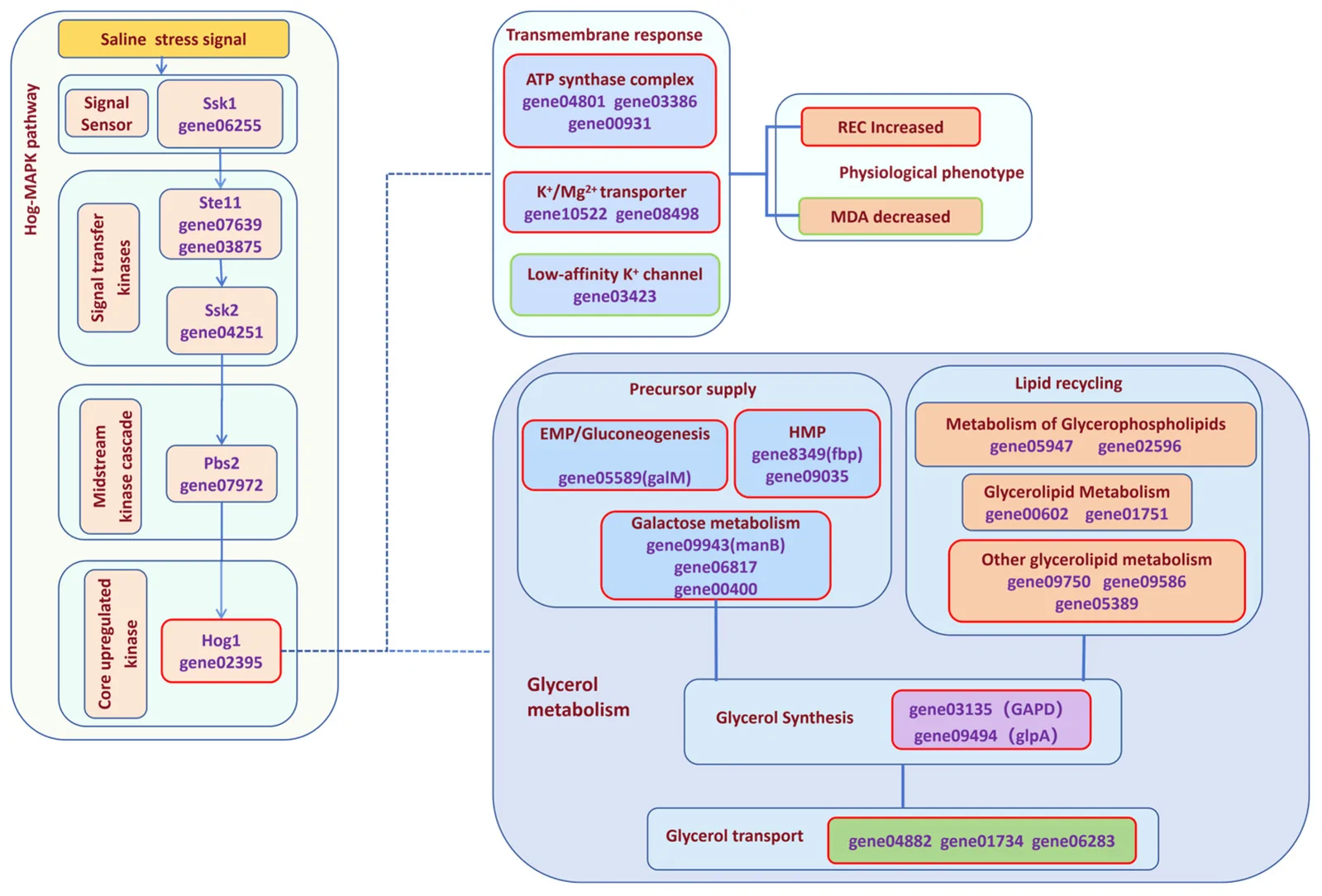
Co-expression network of salt-adaptive genes linked to the HOG-MAPK pathway in *A. westerdijkiae* NDX1.

Overall, extracellular salt stress signals are captured by plasma membrane osmosensors and sequentially relayed through the complete HOG-MAPK signaling cascade. Activated terminal Hog1 synchronously orchestrates three complementary metabolic strategies: 

(1) Rewiring central carbon metabolism and intracellular lipid recycling to supply precursors for *de novo* glycerol production; (2) Inducing high-affinity ion transporters to restore intracellular K^+^/Na^+^ homeostasis; (3) Suppressing low-utilization potassium uptake pathways to save cellular energy. Global transcriptional reprogramming triggered by this cascade ultimately generates the strain’s unique physiological trait: elevated plasma permeability accompanied by suppressed lipid peroxidation under increasing salinity (Figure 10).

The vertical solid cascade on the left represents the canonical conserved HOG signal transduction pathway of filamentous fungi. All grey dashed lines connecting Hog1 to transmembrane transport and glycerol metabolism modules only indicate coordinated transcriptional correlation from RNA-seq data, without confirmed causal regulatory interactions. Red-bordered genes are significantly upregulated DEGs (padj < 0.001, |log_2_FC| ≥ 2); green-bordered genes show reduced expression under 0, 1.0, 1.5 and 2.0 mol/L NaCl stress. The physiological phenotype (elevated REC, decreased MDA) at upper right matches the overall salt-adaptive transcriptional profile. Further gene functional verification is needed to confirm direct regulation between *Hog1* and downstream genes.

## 5. Conclusions

Combining phenotypic observation, physiological measurement and genome-wide transcriptomic profiling, we systematically characterized the salt-responsive molecular features of pickle-isolated *A. westerdijkiae* treated with four gradient NaCl concentrations. A typical non-linear salinity response was observed: mild 1.0 mol/L NaCl significantly promoted mycelial expansion, while high salinity at 2.0 mol/L exerted obvious growth inhibition. Along with elevated NaCl concentration, relative electrical conductivity increased continuously, whereas malondialdehyde content declined gradually, forming a unique physiological phenotype featured by high membrane permeability and attenuated lipid peroxidation under hypersaline environments.

This work fills key research gaps in the field of toxigenic halotolerant food fungi. Distinct from existing transcriptomic research on *A. westerdijkiae* strains isolated from meat and postharvest fruits, the present study combines systematic physiological phenotyping with whole-transcriptome sequencing to dissect the unique salt-adaptive regulatory mechanisms of the strain separated from naturally fermented pickled mustard. A total of 3155 high-confidence salt-responsive DEGs were screened, among which eight core HOG-MAPK cascade genes and 21 glycerol/lipid metabolism-related genes displayed concentration-dependent expression patterns. Transcriptomic evidence revealed coordinated upregulation of lipid catabolic and glycerol synthetic genes, demonstrating that intracellular lipid recycling acts as an auxiliary carbon source to support osmoprotectant glycerol synthesis. All salt-responsive genes identified in this work provide valuable molecular candidates for subsequent gene functional verification and the development of targeted control technologies against ochratoxigenic spoilage fungi in high-salt fermented vegetables.

Nevertheless, this study still possesses several inherent experimental limitations. Firstly, only discrete single time-point samples were collected without continuous dynamic monitoring of salt adaptation processes. Secondly, all metabolic regulatory inferences are solely supported by transcriptomic data, lacking metabolome, proteome or gene knockout/overexpression validation to confirm causal regulatory relationships. Thirdly, all experiments adopted shaken liquid culture systems, which cannot fully replicate the low-oxygen static environment of natural pickling brines. Fourthly, we did not conduct quantitative detection of ochratoxin A under different salt gradients, so quantitative correlations between salinity and mycotoxin production cannot be established based on current data.

In view of the above deficiencies, targeted follow-up research directions are proposed. Time-series multi-omics profiling will be performed to capture dynamic metabolic shifts during long-term salt stress adaptation. Gene knockout and overexpression assays targeting core HOG-MAPK and lipid metabolic genes will be implemented to verify their direct regulatory functions. Independent experiments combining OTA quantitative detection will also be designed to clarify the crosstalk between salt concentration and fungal toxin biosynthesis.

From the perspective of industrial fermented vegetable production, two eco-friendly antifungal strategies are summarized according to our transcriptomic findings. Indigenous lactic acid bacteria and antagonistic yeasts screened from pickling brines can be applied to occupy ecological niches and secrete antifungal metabolites to inhibit the proliferation of *A. westerdijkiae*. Interfering with fungal salt-adaptive pathways via natural bioactive compounds also offers a safe alternative to synthetic chemical fungicides.

Operable preventive suggestions are also put forward for pickle manufacturers and household consumers. Production factories should strengthen raw material cleaning and regular disinfection of processing equipment, as well as reasonably adjust fermentation salinity and temperature to weaken the halotolerance of contaminating fungi. For home producers, pickles need to be stored in sealed refrigerated conditions; any batches with visible mycelium, discoloration or a peculiar odor should be completely discarded. Considering the high thermal stability of OTA and its persistent nephrotoxic and carcinogenic hazards to humans, conventional heating treatment cannot eliminate mycotoxin contamination risk.

## Figures and Tables

**Table 1:** Statistics of transcriptome sequencing data of *A. westerdijkiae* NDX1 under salt stress.

Sample ID	NaCl Treatment (mol/L)	Clean Reads	Total Mapped	Uniquely Mapped	Mapped Genes
NDX0	0	45,596,488	43,309,739 (94.98%)	43,144,375 (94.62%)	9719
NDX1	1	46,303,754	43,665,960 (94.30%)	43,461,059 (93.86%)	9758
NDX1.5	1.5	52,734,312	50,027,804 (94.87%)	49,817,015 (94.47%)	9828
NDX2	2	54,411,292	52,069,777 (95.70%)	51,831,640 (95.26%)	9916

**Table 2:** Information of key genes related to conductivity in *A. westerdijkiae* NDX1 under salt stress.

Gene ID	Functional Module	Key Function Description	KEGG KO Number
gene03423	K^+^ transport module	Low-affinity K^+^ transporter (key for K^+^ uptake)	K20304; K24976
gene03386	Proton pump + membrane homeostasis module	V-ATPase assembly protein (maintains membrane potential)	K23952
gene00931	Proton pump module	Mitochondrial F_1_F_0_ ATP synthase subunit J (energy supply)	K02142
gene04801	Proton pump module	Mitochondrial ATP synthase ε subunit (proton transport assistance)	K02135
gene08498	Other ion transport module	Mg^2+^ transporter (assists ion balance)	K16075
gene10522	K^+^ transport module	K^+^ transporter (clear function, brief annotation)	Unannotated

**Table 3:** Information of key genes related to glycerol metabolism in *A. westerdijkiae* NDX1 under salt stress.

Functional Module		Key Function Description	KEGG KO Number
gene03135	Key catalytic gene	Glycerol-3-phosphate dehydrogenase that directly catalyzes the rate-limiting step of glycerol synthesis	K00006
gene09494 (*glpA*)	Key catalytic gene	Mitochondrial glycerol-3-phosphate dehydrogenase; enhances glycerol synthesis efficiency	K00111
gene08349 (*fbp*)	Precursor supply gene	Fructose-1,6-bisphosphatase, a key glycolytic enzyme; provides glycerol precursor	K01085
gene05589 (*galM*)	Precursor supply gene	Glucose metabolism-related protein; provides a carbon source for glycerol synthesis	K01845
gene00255 (*galM*)	Precursor supply gene	Glucose mutarotase; regulates glucose metabolism toward glycerol synthesis	K01786
gene06802 (*galA*)	Precursor supply gene	Carbohydrate metabolism protein; broadens carbon source utilization	K01207
gene09943 (*manB*)	Precursor supply gene	β-Mannosidase; decomposes mannose to provide a carbon source	K01187
gene06817	Precursor supply gene	Carbohydrate metabolism + transport; regulates carbon source allocation priority	K01845
gene00400	Precursor supply gene	Broad-spectrum carbohydrate metabolism protein; maintains carbon metabolism stability	K01845
gene09035	Precursor supply gene	Regulates carbon metabolism efficiency, prioritizing carbon for glycerol synthesis	K01845
gene00602	Lipid-recycling gene	Lipase 5; decomposes triglycerides to recycle the glycerol backbone	K01046
gene05947	Lipid-recycling gene	Lysophospholipase 1; decomposes phospholipids to supplement glycerol	K01062
gene02596	Lipid-recycling gene	Lysophospholipase 1; cooperates in phospholipid decomposition to improve efficiency	K01062
gene01751	Lipid-recycling gene	Triglyceride decomposition protein; supplements glycerol under carbon deficiency	K01046
gene09750	Lipid-recycling gene	Salt stress-inducible lipid decomposition protein	K01046
gene09586	Lipid-recycling gene	Lipid decomposition auxiliary enzyme; activates lipase activity	K01046
gene05389	Lipid-recycling gene	Lipase precursor ensures long-term lipid decomposition capacity	K01046
gene04882	Transport gene	Putative choline transporter; participates in glycerol extracellular transport	K15646
gene01734	Transport gene	Transmembrane transporter; responsible for glycerol efflux to balance osmotic pressure	K15646
gene06283	Transport gene	Supplementary transporter; stabilizes the glycerol efflux pathway	K15646

Note: Standard homologous gene symbols are shown in parentheses after the corresponding Gene ID. Genes without officially assigned standardized names in *A. westerdijkiae* are indicated by their unique Gene IDs. The functional classification of all genes is presented in the “Functional module” column.

**Table 4:** Information of key genes in the HOG-MAPK signaling pathway of *A. westerdijkiae* NDX1.

Gene ID	Functional Module	Key Function Description	KEGG KO Number
gene02395	MAPK signaling pathway–terminal response	Key: Hog1 kinase regulates downstream stress genes.	K07318
gene07470	MAPK signaling pathway–terminal response	Synergistic Hog1 enhances stress signal.	K07318
gene07972	MAPK signaling pathway–kinase cascade	Bridge kinase activates Hog1.	K07321
gene07639	MAPK signaling pathway–upstream activation	Ste11 activates Pbs2 under mild salt conditions.	K07317
gene03875	MAPK signaling pathway–upstream activation	Ste11 homolog maintains pathway stability.	K07317
gene04251	MAPK signaling pathway–high-salt emergency branch	Ssk2; high-salt-activated MAPKKK.	K07319
gene06255	Signal sensing and transduction—upstream trigger	Salt-sensor histidine kinase.	K07658
gene08338	Signal sensing and transduction—signal stabilization	SAM-domain adaptor; stabilizes signaling.	K07320

**Table 5:** Pearson correlation coefficients between *Hog1* and downstream effector genes.

Downstream Gene	Pearson *r*	*p*-Value	Correlation Degree	Gene Function Description
gene10522	0.8000	0.3333	Strong positive trend	Potassium ion transporter; highly consistent expression trend with Hog1, not significant due to limited sample size
gene09494	0.8000	0.3333	Strong positive trend	*glpA*, a glycerol synthesis gene, upregulated synchronously with *Hog1*
gene08498	1.0000	0.0833	Perfect positive marginal trend	Magnesium ion transporter; expression trend completely consistent with Hog1, approaching a significant level
gene00931	0.8000	0.3333	Strong positive trend	ATP synthase subunit, synchronously upregulated with Hog1
gene03423	−1.0000	0.0833	Perfect negative marginal trend	Low-affinity potassium ion transporter; expression trend completely opposite that of Hog1
gene03135	1.0000	0.0833	Perfect positive marginal trend	*GAPD*, a glycerol synthesis gene, is fully synchronized in expression with *Hog1*
gene04801	0.8000	0.3333	Strong positive trend	ATP synthase subunit with a highly consistent expression pattern with Hog1

## Data Availability

Gene-expression matrix, differentially-expressed gene list and physiological raw data are available at Figshare (DOI: 10.6084/m9.figshare.33188121). Raw transcriptome sequencing reads are deposited in NCBI SRA (under BioProject PRJNA1497641 (accessions: SRR39720775, SRR39720776, SRR39720777, SRR39720778).

## Supplementary Materials

The following supporting information can be downloaded at: https://www.mdpi.com/article/10.3390/microorganisms14081778/s1. Table S1. Primer sequences of selected genes used for qRT-PCR validation. Table S2. PKS/NRPS and secondary metabolism-related differentially expressed genes. Figure S1. GO functional classification of the complete 266 salinity-specific DEGs in 1mol/L NaCl. Figure S2. KEGG enrichment of the 104 low-salt-specific downregulated DEGs in 1 mol/L NaCl. Figure S3. Comparison of expression trends of selected DEGs between qRT-PCR and RNA-seq. Figure S4. Correlation scatter plots of gene expression levels between qRT-PCR and RNA-seq.

## Abbreviations

The following abbreviations are used in this manuscript:

ANOVA	Analysis of variance
ATP	Adenosine triphosphate
cDNA	Complementary deoxyribonucleic acid
CFU	Colony-forming unit
DEGs	Differentially expressed genes
FC	Fold change
FPKM	Fragments per kilobase of transcript per million mapped reads
GO	Gene Ontology
HOG	High-osmolarity glycerol
KEGG	Kyoto Encyclopedia of Genes and Genomes
log_2_FC	Log_2_-transformed fold change
MAPK	Mitogen-activated protein kinase
MDA	Malondialdehyde
NaCl	Sodium chloride
OD	Optical density
OTA	Ochratoxin A
PDA	Potato dextrose agar
PDB	Potato dextrose broth
qRT-PCR	Quantitative real-time polymerase chain reaction
REC	Relative electrical conductivity
RNA-seq	RNA sequencing
SE	Standard error
TBA	Thiobarbituric acid
TCA	Trichloroacetic acid

## References

[1] Hu K., Guo K., Wang X., Li J., Li Q., Zhao N., Liu A., He L., Hu X., Yang Y. (2024). Occurrence of ochratoxin A in Sichuan bacon from different geographical regions and characterization and biocontrol of *Aspergillus westerdijkiae* strain 21G2-1A. Food Res. Int..

[2] Perrone G., Susca A., Cozzi G., Ehrlich K., Varga J., Frisvad J.C., Meijer M., Noonim P., Mahakarnchanakul W., Samson R.A. (2007). Biodiversity of *Aspergillus* species in some important agricultural products. Stud. Mycol..

[3] Hohmann S. (2002). Osmotic stress signaling and osmoadaptation in yeasts. Microbiol. Mol. Biol. Rev..

[4] Turk M., Abramović Z., Plemenitaš A., Gunde-Cimerman N. (2007). Salt stress and plasma-membrane fluidity in selected extremophilic yeasts and yeast-like fungi. FEMS Yeast Res..

[5] Si P., Wang G., Wu W., Hussain S., Guo L., Wu W., Yang Q., Xing F. (2023). SakA Regulates Morphological Development, Ochratoxin A Biosynthesis and Pathogenicity of *Aspergillus westerdijkiae* and the Response to Different Environmental Stresses. Toxins.

[6] Gil-Serna J., Vazquez C., González-Jaén M.T., Patiño B. (2015). Clustered array of ochratoxin A biosynthetic genes in *Aspergillus steynii* and their expression patterns in permissive conditions. Int. J. Food Microbiol..

[7] Rodríguez-Pupo E.C., Pérez-Llano Y., Tinoco-Valencia J.R., Sánchez N.S., Padilla-Garfias F., Calahorra M., Sánchez N.d.C., Sánchez-Reyes A., Rodríguez-Hernández M.d.R., Peña A. (2021). Osmolyte Signatures for the Protection of *Aspergillus sydowii* Cells under Halophilic Conditions and Osmotic Shock. J. Fungi.

[8] Wang Y., Liu F., Pei J., Yan H., Wang Y. (2023). The AwHog1 Transcription Factor Influences the Osmotic Stress Response, Mycelium Growth, OTA Production, and Pathogenicity in *Aspergillus westerdijkiae* fc-1. Toxins.

[9] Plemenitaš A. (2021). Sensing and Responding to Hypersaline Conditions and the HOG Signal Transduction Pathway in Fungi Isolated from Hypersaline Environments: *Hortaea werneckii* and *Wallemia ichthyophaga*. J. Fungi.

[10] Furukawa K., Hoshi Y., Maeda T., Nakajima T., Abe K. (2005). *Aspergillus nidulans* HOG pathway is activated only by two-component signalling pathway in response to osmotic stress. Mol. Microbiol..

[11] Förster C., Marienfeld S., Wendisch F., Krämer R. (1998). Adaptation of the filamentous fungus *Ashbya gossypii* to hyperosmotic stress: Different osmoresponse to NaCl and mannitol stress. Appl. Microbiol. Biotechnol..

[12] Nevoigt E., Stahl U. (1997). Osmoregulation and glycerol metabolism in the yeast *Saccharomyces cerevisiae*. FEMS Microbiol. Rev..

[13] Klein M., Swinnen S., Thevelein J.M., Nevoigt E. (2017). Glycerol metabolism and transport in yeast and fungi: Established knowledge and ambiguities. Environ. Microbiol..

[14] Cui F., Sui N., Duan G., Liu Y., Han Y., Liu S., Wan S., Li G. (2018). Identification of Metabolites and Transcripts Involved in Salt Stress and Recovery in Peanut (*Arachis hypogaea*). Front. Plant Sci..

[15] Ma D., Li R. (2013). Current Understanding of HOG-MAPK Pathway in *Aspergillus fumigatus*. Mycopathologia.

[16] Zeng J., Cao Y., Xu Q., Ran Y., Guo Y., Jiao P., Lang X., Qiao D., Xu H., Cao Y. (2025). The sugar transporter AsSTL is regulated by the kinase Hog1 and is involved in glycerol transport and the response to osmotic stress in the salt-tolerant ascomycete *Aspergillus sydowii* H-1. Fungal Genet. Biol..

[17] Martín J.F., van den Berg M.A., Ver Loren van Themaat E., Liras P. (2019). Sensing and transduction of nutritional and chemical signals in filamentous fungi: Impact on cell development and secondary metabolites biosynthesis. Biotechnol. Adv..

[18] Wang S., Zhou H., Wu J., Han J., Li S., Shao S. (2017). Transcriptomic Analysis Reveals Genes Mediating Salt Tolerance through Calcineurin/CchA-Independent Signaling in *Aspergillus nidulans*. BioMed Res. Int..

[19] He B., Ma L., Hu Z., Li H., Ai M., Long C., Zeng B. (2018). Deep sequencing analysis of transcriptomes in *Aspergillus oryzae* in response to salinity stress. Appl. Microbiol. Biotechnol..

[20] Hapala I., Griac P., Holic R. (2020). Metabolism of Storage Lipids and the Role of Lipid Droplets in the Yeast *Schizosaccharomyces pombe*. Lipids.

[21] Yao S., Zhou R., Jin Y., Zhang L., Huang J., Wu C. (2020). Co-culture with *Tetragenococcus halophilus* changed the response of *Zygosaccharomyces rouxii* to salt stress. Process Biochem..

[22] Sun M., Yao F., Lu L., Shan X., Kong L., Ma Y. (2026). Transcriptomics-based study on the GDP-mannose synthesis pathway and salt tolerance mechanism in *Auricularia heimuer*. Sci. Hortic..

[23] Chen J., Li K., Zhang W., Menghe B. (2024). The antifungal activity of *Lactiplantibacillus plantarum* P9 relates to fatty acid synthesis and purine metabolism-related genes. Food Biosci..

[24] Jiménez-Gómez I., Valdés-Muñoz G., Moreno-Ulloa A., Pérez-Llano Y., Moreno-Perlín T., Silva-Jiménez H., Barreto-Curiel F., Sánchez-Carbente M.d.R., Folch-Mallol J.L., Gunde-Cimerman N. (2022). Surviving in the brine: A multi-omics approach for understanding the physiology of the halophile fungus *Aspergillus sydowii* at saturated NaCl concentration. Front. Microbiol..

[25] Ariño J., Ramos J., Sychrová H. (2010). Alkali metal cation transport and homeostasis in yeasts. Microbiol. Mol. Biol. Rev..

[26] Liu Y., Lu J., Cui L., Tang Z., Ci D., Zou X., Zhang X., Yu X., Wang Y., Si T. (2023). The multifaceted roles of Arbuscular Mycorrhizal Fungi in peanut (*Arachis hypogaea*) responses to salt, drought, and cold stress. BMC Plant Biol..

[27] Avis T.J., Michaud M., Tweddell R.J. (2007). Role of lipid composition and lipid peroxidation in the sensitivity of fungal plant pathogens to aluminum chloride and sodium metabisulfite. Appl. Environ. Microbiol..

[28] Vela-Corcía D., Bautista R., de Vicente A., Spanu P.D., Pérez-García A. (2016). *De novo* Analysis of the Epiphytic Transcriptome of *Podosphaera xanthii* and Identification of Candidate Secreted Effector Proteins. PLoS ONE.

[29] Corchete L.A., Rojas E.A., Alonso-López D., De Las Rivas J., Gutiérrez N.C., Burguillo F.J. (2020). Systematic comparison and assessment of RNA-seq procedures for gene expression quantitative analysis. Sci. Rep..

[30] Kim D., Langmead B., Salzberg S.L. (2015). HISAT: A fast spliced aligner with low memory requirements. Nat. Methods.

[31] Li B., Dewey C.N. (2011). RSEM: Accurate transcript quantification from RNA-Seq data with or without a reference genome. BMC Bioinform..

[32] Jones L., Riaz S., Morales-Cruz A., Amrine K.C.H., McGuire B., Gubler W., Walker M.A., Cantu D. (2014). Adaptive genomic structural variation in the grape powdery mildew pathogen, *Erysiphe necator*. BMC Genom..

[33] Livak K.J., Schmittgen T.D. (2001). Analysis of relative gene expression data using real-time quantitative PCR and the 2^−ΔΔCt^ method. Methods.

[34] Jiménez-Gómez I., Valdés-Muñoz G., Moreno-Perlín T., Mouriño-Pérez R.R., Sánchez-Carbente M.d.R., Folch-Mallol J.L., Pérez-Llano Y., Gunde-Cimerman N., Sánchez N.d.C., Batista-García R.A. (2020). Haloadaptive Responses of *Aspergillus sydowii* to Extreme Water Deprivation: Morphology, Compatible Solutes, and Oxidative Stress at NaCl Saturation. J. Fungi.

[35] Pérez-Llano Y., Rodríguez-Pupo E.C., Druzhinina I.S., Chenthamara K., Cai F., Gunde-Cimerman N., Zalar P., Gostinčar C., Kostanjšek R., Folch-Mallol J.L. (2020). Stress reshapes the physiological response of halophile fungi to salinity. Cells.

[36] Pérez-Llano Y., Fernando L.D., Dickwella Widanage M.C., Jacob A., Martínez-Ávila L., Lipton A.S., Gunde-Cimerman N., Latgé J.P., Batista-García R.A., Wang T. (2023). Structural adaptation of fungal cell wall in hypersaline environment. Nat. Commun..

