# Supplementary material for: Transcriptomic Analysis Insights into Salt Adaptation of Pickle-Derived Aspergillus westerdijkiae
Source: Microorganisms. 2026 Aug 12;14(8):1778. doi: 10.3390/microorganisms14081778 (PMC13515104; doi:10.3390/microorganisms14081778)
Supplement: Supplementary file 1 [file microorganisms-14-01778-s001.zip › microorganisms-4421564-supplementary.pdf]

Supplementary Table S1 Primer sequences of selected genes used for qRT-PCR validation

Gene ID	**Forward**	**Reverse**	**Amplicon length**
gene05555	GACTACCAACTTGCCACATCAACAG	AAGAACAAAGCCAGACCCTCTCTC	123
gene06971	CCTCCTCGTTCGCTGCCATC	AGCTGATCGGATTCCAGTCTTCTTC	87
gene10399	TGGGACTGGGCTAAGAGGTATTCG	ACAGGTCGGACGGCGGTATC	114
gene10279	GTCTAGTGAACGAGGAACGCTTGG	GATTGGCTGCGGAAGGCTGTC	107
gene08478	GTCCTGTCCCGCATACATTCCTTC	TCCTCGTCAGCCGCTTCCTC	146
gene07639	AGCCTGTCACGCAATTCTGTCTC	GCCACCACCGCTGCTTGATAG	103
gene06402	TGCGATGAGGAGGAGCCCAAG	TTCCGAGTCCAGGTCAGAGAAGC	150
gene03761	GACTGGTTGGACTGGAAGGTTGTG	GCGGATTTCGGAGTTGGTGAGAG	123
gene04258	TAATCCCAACGCTGAAA	GCACTTACGACCCGAAT	298
gene04434	GTGGCGGATAAGCAGAA	GGGAATGATAGCGATGAAG	165
gene08992	CAACAATGCCCAACGAGG	AGGACGAGCACGAAACCC	168
gene09590	GGCTCTTTGAGTATTTGCTT	TTCTTCTGAGACGCCTTGT	177
gene04253	TACCTCACCTGCTCCGCTATCTTC	TCTGGTTCTTGCTCTGGATGTTGC	88

Supplementary Table S2: PKS/NRPS and secondary metabolism-related differentially expressed genes

Category	Up (↑)	Down (↓)
**PKS**	gene05546, gene05978	gene00118, gene00183, gene00841, gene01616, gene01906, gene02399, gene02654, gene09768, gene10058
**NRPS**	gene07371	gene02923, gene07073
**PKS-NRPS**	gene07869	gene01828
**Methyltransferase**	gene04697, gene06753, gene06848	gene10059
**Prenyltransferase**	-	gene00469, gene03961, gene05200
**P450**	gene02759	gene08881
**Halogenase**	gene01830	-
**Dioxygenase**	-	gene03429, gene09019
**Other oxidoreductases**	gene02928, gene09984	gene01618, gene10060, gene10061

**Acetyltransferase**	gene06973	-
**Transcription factor**	gene04488, gene09133	-

Notes: ↑/↓, significant up/downregulation (adj. $P < 0.05$); PKS, polyketide synthase; NRPS, non-ribosomal peptide synthetase; functions from GO/KO.

Supplementary Figure S1: GO functional classification of the complete 266 salinity-specific DEGs in 1mol/L NaCl

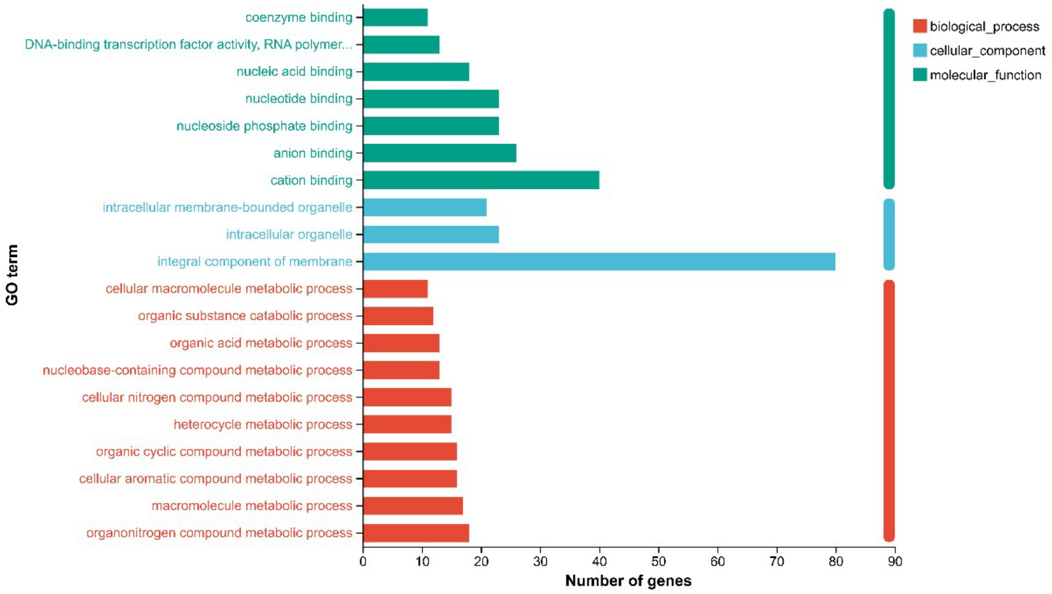

Y-axis: enriched GO terms separated into biological process (red), cellular component (blue), molecular function (green); X-axis: the count of enriched DEGs per GO term.

Supplementary Figure S2：KEGG enrichment of the 104 low-salt-specific downregulated DEGs in 1mol/L NaCl

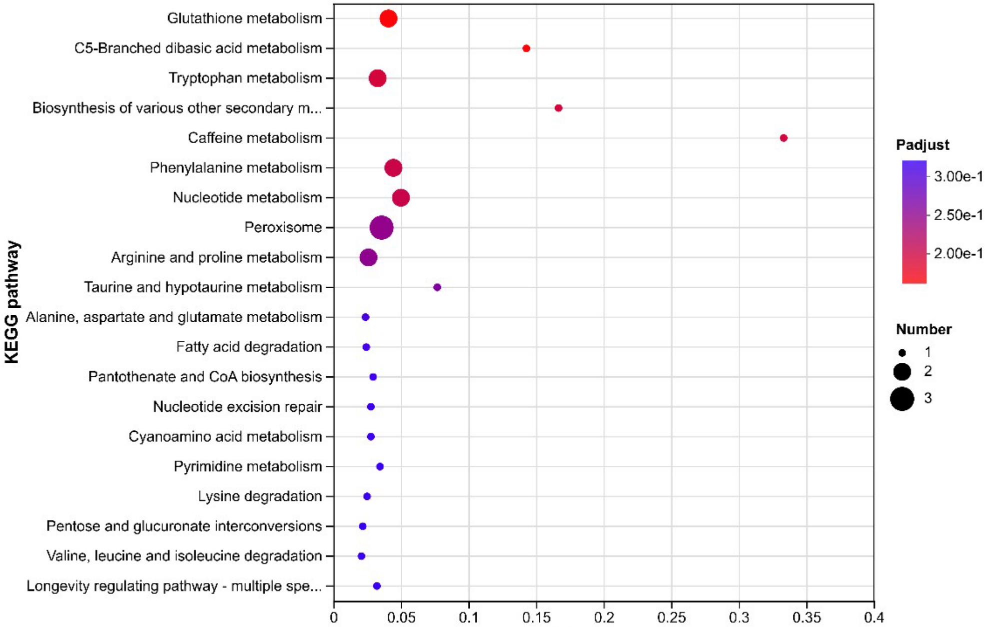

X-axis: enrichment score; Y-axis: KEGG functional pathways. Bubble size reflects the count of enriched DEGs; bubble color represents adjusted p-value (padj).

Supplementary Figure S3: Comparison of expression trends of selected DEGs between qRT-PCR and RNA-seq

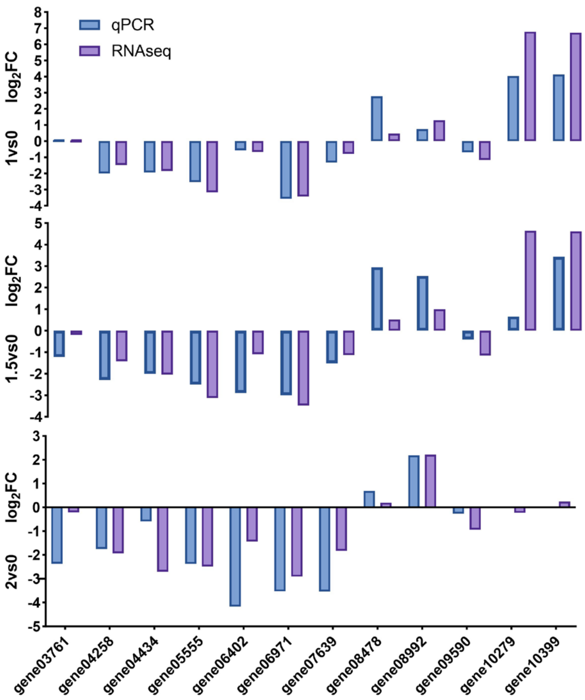

The x-axis shows randomly selected DEG IDs, and the y-axis represents log_2-transformed fold change relative to the 0 mol/L control. Blue bars indicate qRT-PCR results, and purple bars indicate RNA-seq results. Consistent expression trends confirmed the reliability of the transcriptome data.

Supplementary Figure S4: Correlation scatter plots of gene expression levels between qRT-PCR and RNA-seq

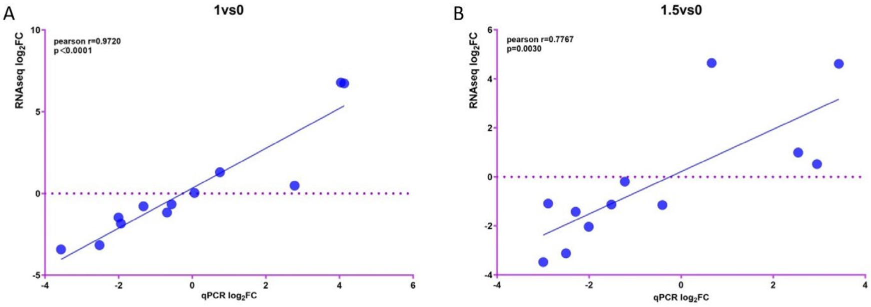

The x-axis represents log_2-transformed fold change from qRT-PCR, and the y-axis represents log_2-transformed fold change from RNA-seq. A–D correspond to 1.0 vs 0, 1.5 vs 0, 2.0 vs 0 mol/L comparisons, and the combined total dataset, respectively. Pearson correlation coefficients and *P* values are shown.
